# Muscle macrophage regenerative response after squalene-adjuvanted influenza vaccination drives Th2-skewed response and is reduced with age

**DOI:** 10.21203/rs.3.rs-5760877/v1

**Published:** 2025-01-28

**Authors:** Moataz Noureddine, Lauren A. Chang, Farah El Ayache, Gabriel Laghlali, Eleanor Burgess, Leonie Gruneberg, Prajakta Warang, Kaijun Jiang, Haye Nijhuis, Lynda Coughlan, Juan Garcia-Bernalt Diego, Seokchan Park, Jorge Levican, Michael Schotsaert

**Affiliations:** 1 Department of Microbiology, Icahn School of Medicine at Mount Sinai, New York, NY, 10029, USA; 2 Global Health and Emerging Pathogens Institute, Icahn School of Medicine at Mount Sinai, New York, NY, 10029, USA; 3 Graduate School of Biomedical Sciences, Icahn School of Medicine at Mount Sinai, New York, NY 10029, USA; 4 Department of Pharmaceutics, Ghent University, Ghent, Belgium; 5 Department of Microbiology and Immunology, University of Maryland School of Medicine, Baltimore, MD 21201, USA; 6 Center for Vaccine Development and Global Health (CVD), University of Maryland School of Medicine, Baltimore, MD 21201, USA; 7 Icahn Genomics Institute, Icahn School of Medicine at Mount Sinai, New York, NY, 10029, USA; 8 Marc and Jennifer Lipschultz Precision Immunology Institute, Icahn School of Medicine at Mount Sinai, New York, NY, 10029, USA

**Keywords:** Vaccination, Muscle regeneration, Aging, Influenza, Macrophages, Tregs

## Abstract

Squalene-based adjuvants like MF59, and its research alternative AddaVax, induce transient muscle injury, but their working mechanisms downstream of muscle injury remain unclear. We show that an AddaVax-adjuvanted quadrivalent inactivated influenza virus vaccine (QIV) intramuscular injection triggers muscle regeneration-like immune processes and increases CX3CR1^+^Ly6C^+^ macrophages in the muscle and inguinal lymph nodes by day 4 post-injection. This leads to a Th2 skewed vaccine response with higher levels of vaccine specific IgG1 titers, and Th2-associated cytokines in the lungs 5 days after subsequent influenza viral challenge. In aged mice, the macrophage recruitment and polarization is diminished, which is consistent with age-associated muscle mass loss, reflecting the age-related decline in muscle regeneration. Unlike young mice, aged mice exhibit a reduction in magnitude and skewing of AddaVax-mediated Th2 responses to QIV. We found that adoptive transfer of bone marrow-derived macrophages derived from young mice into aged mice at the moment of vaccination leads to their infiltration into the injected muscle, where they collect vaccine antigens, drain to the lymph node, and enhance the Th2 response, recapitulating the young host response but in an older host. However, rescuing the Th2-skewing effects of AddaVax alone was not sufficient to enhance protection against mismatched subsequent influenza viral infection in aged mice, suggesting additional factors at play in the diminished vaccine response in aged hosts. This underscores the importance of the macrophage-driven muscle regenerative response in the mechanism of action for squalene-based adjuvants like AddaVax and emphasizes the need to study how muscle damage and regenerative pathways in intramuscular vaccine responses contribute to vaccine effectiveness.

## Summary

Squalene-adjuvanted influenza vaccine efficacy and adjuvant-mediated type 2 immunity depend on the muscle macrophage responses resembling muscle regeneration upon injury, and is impaired by the age-associated decline of macrophage responses in the muscle post-injection.

## Introduction

Seasonal influenza has a severe public health impact, causing hundreds of thousands of deaths and millions of hospitalizations globally every year (Javanian et al., 2021). Currently, there are no effective antiviral drugs to treat patients with severe influenza infections, so the primary clinical approach is preventive using seasonal vaccines, such as quadrivalent inactivated influenza vaccines (QIV) (Gaitonde, 2019). People living with advanced age are disproportionately affected during influenza virus infection. Current influenza vaccine formulations, including the high-dose and MF59-adjuvanted vaccines designed to improve efficacy in the geriatric population, fail to curb the exponential death rates seen in aged individuals (Hansen et al., 2020). According to the CDC, although around 70% of individuals aged 65 and older receive QIV every year in the US, this group still accounts for over 75% of total influenza-associated deaths. This has prompted extensive and ongoing research aimed at developing novel adjuvants and better understanding the mechanism of MF59 to further improve vaccine efficacy in this vulnerable population (Lu et al., 2019; Machado et al., 2021). The mechanism of action for MF59 remains unknown; however, it has been shown to recruit a variety of immune cells like eosinophils and macrophages to the injected muscle, and to depend on transient muscle injury, including ATP release as a damage-associated molecular pattern (DAMP). MF59 leads to a Th2-skewed response to vaccines (Ko & Kang, 2018; Lampe et al., 2020; Nian et al., 2021; Vono et al., 2013). Despite the association of MF59’s mechanism to transient muscle injury, it remains unclear whether the muscle regenerative pathway is activated following MF59 injection, whether it overlaps with the adjuvant-driven vaccine response, or if muscle regeneration plays a role in MF59’s mechanism of action in adjuvanting vaccine responses (Calabro et al., 2011; Mosca et al., 2008; van Aalst et al., 2017). This gap in knowledge was partly due to the previously limited characterization of the regenerative response following injection-induced muscle damage post-vaccination. However, recent research outside of the realm of vaccination has provided a more comprehensive characterization, describing post-transient injury muscle regeneration as a conserved immune response that initiates with a pro-inflammatory stage and concludes with a late pro-repair phase with complete muscle repair (W. Yang & Hu, 2018).

The pro-inflammatory stage peaks 2 days post-injury and is marked by an influx of neutrophils, eosinophils, CD8 T cells, and iNOS^+^ pro-inflammatory muscle macrophages that express CX3CR1, are derived from monocytic origins, and express Ly6C (Chazaud, 2020). This initial environment is rich in IFNγ and TNFα (Babaeijandaghi et al., 2022), which function as essential signaling molecules that activate myocyte progenitor cells. By day 4, the response is largely replaced by a pro-resolution or pro-repair response characterized by the influx of Tregs which repolarize macrophages towards an M2 anti-inflammatory phenotype expressing Arginase I and anti-inflammatory cytokines like IL-4 and IL-10 (Heredia et al., 2013). The anti-inflammatory milieu then allows for the proliferated progenitors to differentiate into fully functional myocytes. This process when homeostatic allows complete muscle regeneration, such as for expected and frequent injuries occurring due to motor movement. Muscle regulatory T cells (Tregs) have also been shown to accumulate in the draining lymph nodes in the repair process (Burzyn et al., 2013; Tidball, 2017).

The muscle regenerative pathway has been shown to be largely impaired with age. The decline in muscle regeneration has been attributed to age-associated macrophage senescence and to muscle chronic inflammation (Kunz & Lanza, 2023; Sousa et al., 2023). The late pro-repair stage appears to be the most affected by aging including pro-repair macrophages and Tregs (Kuswanto et al., 2016; Sousa et al., 2024; Wu et al., 2022). Given that MF59’s mechanism of action may depend on muscle regeneration and elderly individuals are the clinical group receiving MF59-adjuvanted QIV, we sought to understand the impact of age and muscle regeneration on MF59-adjuvanted QIV effectiveness. Our findings demonstrate that the adaptive immune response following an AddaVax- (research-grade MF59) adjuvanted quadrivalent influenza vaccine (aQIV) injection in 6- to 8-week-old (young) mice is skewed towards a Th2 response. We also show that both the pro-inflammatory and pro-repair stages of the regenerative pathway occur after administration of AddaVax alone or aQIV. In draining lymph nodes, the pro-repair stage is reflected with an increase in CX3CR1^+^Ly6C^+^ macrophages and Tregs only following an aQIV intramuscular injection, but not AddaVax alone, coinciding with the onset of the B-cell response. Furthermore, the immune kinetics in 18- to 24-month-old (aged) mice are impaired, with a reduction in pro-repair macrophage and Treg responses in the muscle and draining lymph nodes, as well as a decreased Th2 skewed response. Finally, we show that this Th2 skewing can be rescued by the upstream macrophage response in the muscle post-vaccination. Specifically, injecting aged mice intravenously with bone marrow-derived macrophages from the femurs of young mice (yBMDM) one day prior to aQIV vaccination restores the Th2 response seen in younger mice. This intervention did not rejuvenate the vaccine humoral response, and didn’t significantly reduce lung viral load after a subsequent Influenza challenge highlighting the importance of investigating aging consequences on other immune cells like B-cells.

## Results

### AddaVax skews QIV response towards a Th2 response and enhances protection against lethal Influenza challenge in 6–8-week-old mice.

**Fig 1.**
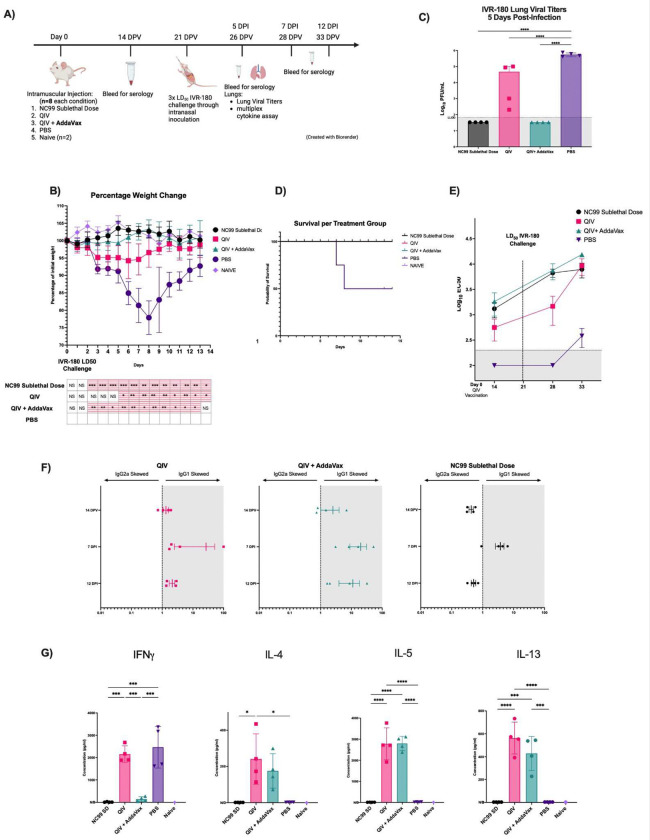
AddaVax-adjuvanted QIV shows enhanced protection and shares a Th2-skewed response to subsequent influenza lethal challenge compared to unadjuvanted QIV. (A) Experimental schematic. (B) Percentage weight loss after 3LD_50_ IVR-180 viral challenge. The table below graph has filled boxes to indicate a day where the group is significantly different from the PBS group. (C) Bar graph of lung viral titers 5 days post-infection measured by plaque assay. (D) Survival plot after 3LD_50_ IVR-180 viral challenge. (E) Total vaccine specific IgG titers at 14-, 28- and 33-days post-vaccination (5 days post-challenge) measured by ELISA. (F) Plots showing IgG ELISA isotype subclass IgG1:IgG2a titers ratio at at 14-, 28- and 33-days post-vaccination (5 days post-challenge) (G) Bar plots of lung cytokines measured using a multiplex cytokine assay 5 days post-challenge with 3LD_50_ IVR-180 . * P ≤ 0.05 ** P ≤ 0.01 *** P ≤ 0.001 **** P ≤ 0.0001 (one-way ANOVA).

AddaVax has been shown to enhance Th2 responses when supplemented with a variety of vaccines (Nian et al., 2021). To investigate whether AddaVax has a similar immune-skewing effect on QIV, we vaccinated mice with the Fluzone High-Dose^®^ (2021–2022) seasonal quadrivalent inactivated flu vaccine with no adjuvant (QIV), AddaVax-adjuvanted aQIV, or PBS as a negative control. One group received a sublethal dose of H1N1 influenza A virus A/New Caledonia/20/99 (NC99) on the day of vaccination as a positive control for a Th1 antiviral response, and to simulate pre-existing immunity (Fig. 1A). NC99 was chosen since this is an H1N1 influenza virus that circulated before the 2009 H1N1 pandemic and was included as a vaccine strain in the influenza vaccines until the 2005–2006 season (Choi et al., 2020). Twenty-one days after vaccination, all groups were challenged with a lethal dose of IVR-180 (A/Singapore/gp1908/2015). We used a vaccine-mismatched H1N1 viral strain to model the vaccine’s performance specifically during a season when circulating strains do not antigenically match the administered seasonal vaccine completely. We chose this scenario because the chances of a mismatch occurring are up to six times more common than having a complete match between circulating strains and the administered seasonal flu vaccine (Chan et al., 2018). The NC99 and aQIV groups showed no weight loss post-infection, while the QIV group lost body weight, reflecting morbidity. The QIV and PBS groups lost weight similarly for the first 5 days post-viral challenge with a minimum average of 95% (SD = 5%) for the QIV group (Fig. 1B). Due to the observed variability of weight loss per group 5 days post-infection, lungs were collected on that day to evaluate the groups ability to control viral infection and to characterize their lung immune response to infection. Five days post-infection, no viral titers were detected in the lung homogenates of the NC99 and aQIV groups (Fig. 1C). The QIV group showed detectable viral titers, with a significant decrease compared to the PBS group. Only the PBS group showed a 50% probability of mortality by day 10 post-viral challenge and a minimum average weight of 77.8% (SD = 4.3%) of original weight 8 days after viral challenge (Fig. 1D). These findings demonstrate that unadjuvanted QIV provides protection against influenza-associated mortality in young mice. The addition of AddaVax allows for earlier, robust control over viral titers with no detectable morbidity, similar to the NC99 group where immunity is acquired through a previous sublethal infection.

To characterize the type of immune response enhanced by the addition of AddaVax to QIV, we assessed the vaccine-specific antibody levels by collecting serum at 14-, 28-, and 33-days post-vaccination and quantifying vaccine-specific IgG1, IgG2a, and total IgG titers. We found significantly higher vaccine-specific total IgG titers in mice that received aQIV (3-fold increase) and the NC99 sublethal dose (2-fold increase) compared to QIV alone at 12 days after vaccination correlating with viral persistence in the lungs after influenza virus challenge. Vaccine-specific IgG titers in the QIV group reached the levels of the aQIV and NC99 groups by day 12 after infection, unlike the PBS group, where vaccine-specific antibodies became detectable but 28-fold lower than the QIV group (Fig. 1E).

14 days after vaccination, the higher vaccine-specific antibody levels observed with the addition of AddaVax consisted mainly of an increase in the IgG1 subtype, as indicated by a higher IgG1:IgG2a ratio in the aQIV group (ratio of 2.5:1). This differed from the QIV group, where the response was 1.3:1 ratio, and from the NC99 group, where, IgG2a was the dominant antibody subtype (ratio of 1:2.3). Antibody isotype skewing towards IgG1 is indicative of a Th2-biased response (Germann et al., 1993; Snapper & Paul, 1987; Stevens et al., 1988). The cytokine composition of the lung homogenates corroborated our antibody analyses. We found that adjuvanting QIV with AddaVax resulted in a type 2-skewed host response to infection, with hallmark type 2 cytokines IL-4, IL-5, and IL-13 present in the lung homogenates of the aQIV group at 5 days post-infection. Interestingly, the unadjuvanted QIV group also displayed a Th2-associated cytokine profile, but differed from aQIV with the presence of IFNγ (Fig. 1G). Presence of IFNγ and persistence of viral load in the lungs highlight the potential for a *de novo* and not a recall Th1 response in the unadjuvanted QIV group, which was virtually absent in the aQIV group (Koutsakos et al., 2023). This is supported by our previous research showing that unadjuvanted QIV is a poor inducer of T-helper cell responses and that intramuscular vaccination with QIV skews host responses to infection towards type 2 (Jangra et al., 2022). QIV also showed higher cytokine levels associated with influenza-induced inflammation, such as IL-1β, TNFα, IL-2, and IL-6, similar to the PBS group, which did not control viral replication well (Fig. S1A–E). We also detected higher levels of IL-18 and IL-12p70 in the QIV and PBS groups (Fig. S1F,G). Furthermore, we only found detectable levels of circulating IFNγ^+^ T-cells 14 days after vaccination after antigen-specific restimulation (cocktail of H1N1 HA peptide, IVR-180 virus, and influenza A virus nucleoprotein peptide) or general activation with phorbol 12-myristate 13-acetate (PMA) and ionomycin, in the NC99 group alone, but not in any of the QIV-vaccinated groups or PBS control group (Fig. S1H,I). These findings demonstrate that the aQIV induces a Th2 response which in turn provides better protection against a lethal Influenza infection compared to QIV alone.

### AddaVax induces an early pro-inflammatory regenerative response in the muscle which is partially mirrored in the draining inguinal lymph nodes upon addition of QIV.

Next, we aimed to investigate the potential mechanisms behind how AddaVax skews the vaccine response for QIV towards Th2. Currently, the mechanism of action of AddaVax and its clinical derivative, MF59, is not fully understood. Previous research has shown that AddaVax induces muscle damage, which potentiates its adjuvant effect (Kim et al., 2020; Vono et al., 2013). Since muscle damage is followed by muscle regeneration in healthy hosts, this suggests that muscle regeneration could occur after an MF59-adjuvanted vaccination.

To demonstrate that AddaVax triggers muscle regeneration, we injected the quadriceps muscles of mice with PBS, AddaVax, QIV, and aQIV. This approach allowed us to observe the contributions of QIV and AddaVax injections to muscle response separately and in combination. We harvested the muscles on days 2 and 4 to investigate if both the pro-inflammatory and anti-inflammatory responses that make up the regenerative process were induced. We also collected the draining inguinal lymph nodes to determine whether changes in the muscle were mirrored in the lymph nodes. We utilized two spectral flow cytometry panels that enabled us to characterize more than ten different immune populations in both organs. Additionally, we labeled all circulating immune cells by intravenous injection of fluorochrome-conjugated anti-CD45 to exclude non-tissue infiltrating immune cells (Anderson et al., 2014) (Fig. S2 and S3).

**Fig 2.**
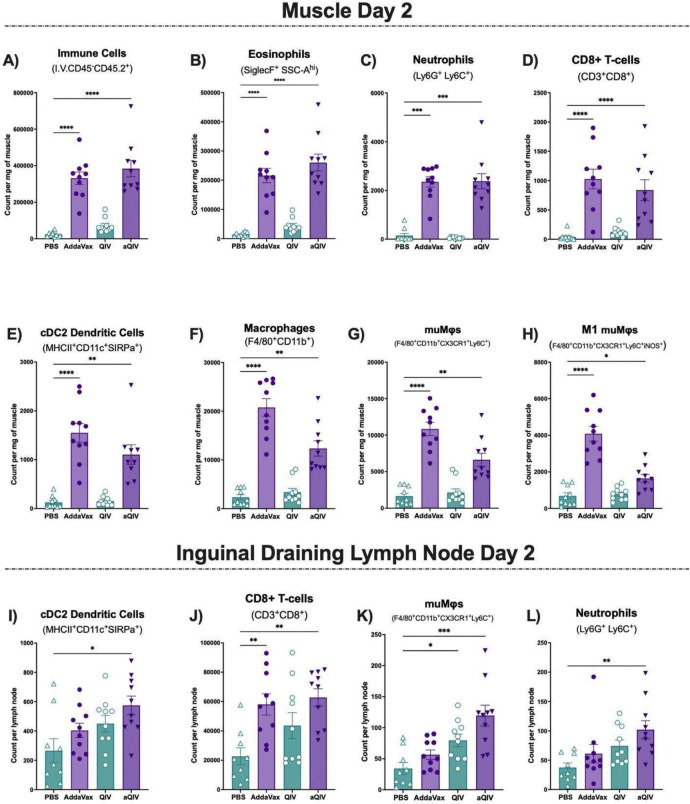
Day 2 intramuscular and draining inguinal lymph node immune kinetics post-vaccination with AddaVax, QIV and aQIV compared to PBS. Count of non-circulating **(A)** immune cells, **(B)** eosinophils, **(C)** neutrophils, **(D)** CD8^+^ T-cells, **(E)** cDC2 dendritic cells, **(F)** macrophages, **(G)** muMφs and **(H)** M1 muMφs in the quadriceps muscle 2 days after PBS, AddaVax, QIV, or aQIV injection. Count of non-circulating **(I)** cDC2 dendritic cells, **(J)** CD8^+^ T-cells, **(K)** muMφs and **(L)** neutrophils in the draining inguinal lymph nodes 2 days after intramuscular PBS, AddaVax, QIV and aQIV injections. * P ≤ 0.05 ** P ≤ 0.01 *** P ≤ 0.001 **** P ≤ 0.0001 (Kruskal-Wallis test).

On day 2, we found a regenerative-like pro-inflammatory response in muscles of the AddaVax and aQIV groups, but not in the QIV or PBS groups. There was a 14-fold and 16-fold increase in the count of immune cells per milligram of muscle in the AddaVax and aQIV groups respectively compared to the PBS-injected muscles (Fig. 2A). The immune response to QIV was significantly lower than the AddaVax injected muscles, with a 3-fold increase compared to the PBS-injected muscles. The immune cell increase in the AddaVax and aQIV groups was driven by an increase of neutrophils, eosinophils, and CD8 T-cells counts per milligram of muscle (Fig. 2B–D). The eosinophil count per milligram of muscle increased by 15-fold in the AddaVax group, 3-fold in the QIV group, and 19-fold in the aQIV group compared to the PBS control group. The neutrophil increase was identical between the AddaVax and aQIV groups with a 16-fold increase, meanwhile the QIV group showed no neutrophil increase compared to the PBS group. We also observed an increase in dendritic cell counts per milligram of muscle (Fig. S4A), particularly in cDC2s in the AddaVax and aQIV groups only, where they increased by 12- and 9-fold respectively compared to the PBS group (Fig. 2E). CX3CR1^+^Ly6C^+^ macrophages or muscle-associated macrophages (muMφs) (Fig. 2F) that were phenotypically pro-inflammatory, as indicated by iNOS expression, increased by 6- and 2-fold in the AddaVax and aQIV groups respectively and no increase in the QIV group compared to the PBS negative control group (Fig. 2G) (Xue et al., 2018). Early proinflammatory muMφs are crucial in initiating the inflammatory response to damage and prepare the niche for regeneration by clearing debris and stimulating the proliferation of myocyte progenitors (Dort et al., 2019). These findings show that AddaVax alone can induce a pro-inflammatory regenerative response on day 2, regardless of the presence of QIV. Interestingly, the addition of QIV reduced the macrophage response by half, while the dendritic cell response was independent of QIV and required AddaVax.

It is unknown whether proinflammatory immune cells mediating muscle regenerative responses drain to the lymph nodes at early time points, so we also investigated the draining lymph nodes. On day 2, we observed a significant increase in cDC2s in the draining inguinal lymph nodes of the aQIV group alone (2-fold increase compared to PBS) (Fig. 2I). No such increase was present for the AddaVax group or QIV group. This indicates that the presence of a dendritic cell recruitment in the muscle is dependent on AddaVax, but drainage to the inguinal lymph nodes may rely on the presence of QIV at the injection site. We also found an AddaVax-dependent increase in CD8 T-cells in the draining lymph nodes of both the AddaVax and aQIV groups, similar to the AddaVax and aQIV injected muscles (Fig. 2J). Regarding the macrophage response, we observed an increase in muMφs in both the QIV (2-fold) and aQIV groups (4-fold) over the PBS negative control group (Fig. 2K). It is possible that the reduction in this macrophage population with the addition of QIV to AddaVax in the muscle at the same time point compared to AddaVax alone is indicative of drainage to the lymph nodes. Additionally, we observed an increase in neutrophils in the aQIV group, which has been previously noted and shown to be dispensable in the MF59-adjuvanted vaccine response (Calabro et al., 2011). These findings reveal a regenerative-like pro-inflammatory milieu present in the muscle 2 days post-injection with AddaVax or aQIV consists of eosinophils, neutrophils, proinflammatory macrophages and CD8 T-cells, and correlates with a robust dendritic cell recruitment. In the aQIV group alone, the inflammatory cells are partially mirrored in the draining lymph nodes with an increase in neutrophils, muMφs, CD8 T-cells and cDC2s.

### aQIV induces the late-stage pro-repair regenerative response in the muscle and draining inguinal lymph nodes overlapping with the start of the B cell response.

The late-stage pro-repair response in muscle regeneration has been shown to involve pro-repair macrophages that have been repolarized by infiltrating Tregs to an anti-inflammatory phenotype. This typically occurs at day 4 post-damage. The pro-repair macrophages secrete IL-4, IL-10 and TGFβ to promote wound healing (Wynn & Vannella, 2016). They also express CX3CR1 on their surface, and express variable levels of Ly6C. These pro-repair macrophages also lack iNOS expression and express variable levels of Arginase I (Tidball et al., 2014; Wculek et al., 2022). IL-4 and IL-10 have been implicated in polarizing T-cells towards a Th2 response and inhibiting a Th1 response (Fallon et al., 2002; Laouini et al., 2003). Given this existing pro-repair muscle response following damage, we hypothesized that monocyte-derived macrophages infiltrate the AddaVax-injected muscle after injection and are eventually polarized toward a pro-repair phenotype and secrete IL-4 and IL-10. These macrophages then assist in the local wound healing process and, in conjunction with Tregs, polarize the QIV-specific T-cell response toward a Th2 response in the draining lymph nodes by expressing Th2-inducing cytokines such as IL-4 and IL-10. To investigate this hypothesis, we injected the quadriceps of mice with PBS, AddaVax, QIV, and aQIV. We collected the injected muscles and the inguinal draining lymph nodes on day 4 and characterized the immune composition using spectral flow cytometry.

**Fig 3.**
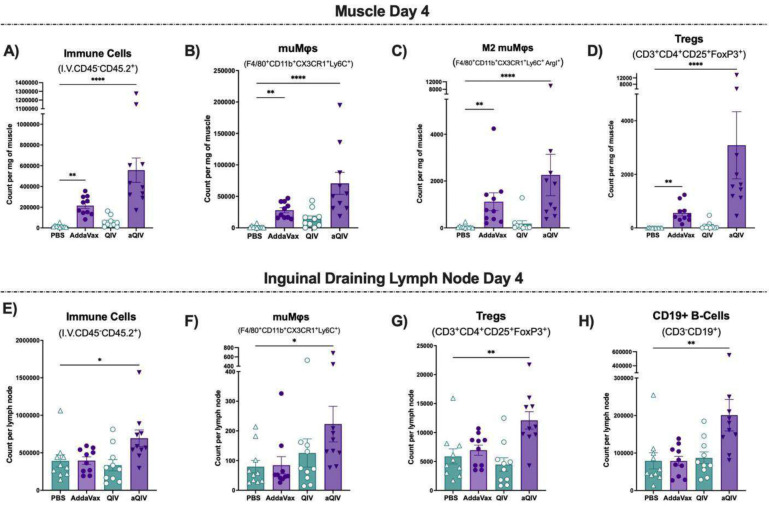
Day 4 intramuscular and draining inguinal lymph node immune kinetics post-vaccination with AddaVax, QIV and aQIV compared to PBS. Count of non-circulating **(A)** immune cells, **(B)** muMφs, **(C)** M2 muMφs and **(D)** Tregs in the muscle at day 4. Count of non-circulating **(E)** immune cells, **(F)** muMφs, **(G)** Tregs and **(H)** B-cells in the inguinal draining lymph nodes 4 days after PBS, AddaVax, QIV, or aQIV injection. * P ≤ 0.05 ** P ≤ 0.01 *** P ≤ 0.001 **** P ≤ 0.0001 (Kruskal-Wallis test).

On day 4, the number of infiltrated immune cells per milligram remained elevated in the AddaVax and aQIV groups, similar to day 2, but compositionally shifted (Fig. 3A). There was a 3-fold decrease in eosinophils per milligram of muscle in the AddaVax group and a 2-fold decrease in the aQIV group compared to day 2 (Fig. S4F and S4G). muMφs persisted at the same numbers in both the AddaVax and aQIV groups, with a count that was 11-fold higher in the aQIV group and 3-fold higher in the AddaVax group compared to day 2, now comprising a larger percentage of the total immune cells present in the muscle on day 4 (Fig. 3B, S4I). Unlike at day 2, a subset of the muMφs expressed Arginase I on day 4 in the AddaVax and aQIV groups, indicating a shift towards an anti-inflammatory phenotype (Fig. 3C and S4E). iNOS-expressing muMφs and neutrophils persisted at similar counts as day 2 (Fig. S4G and S4H), showing that the pro-inflammatory milieu was subsiding but not completely resolved by day 4.

We also found an increase in Treg count per milligram of muscle in the AddaVax and aQIV groups compared to PBS, with an 8-fold increase of Tregs in the aQIV-injected muscles compared to day 2 (Fig. 3E and S4D). The total T-cell population dropped by half in the AddaVax-injected muscles by day 4 but increased 2-fold in the aQIV group, suggesting that the T-cell numbers increase due to AddaVax but persist for longer in the presence of QIV (Fig. S4B). The overall increase in the T-cell population was driven by a 3-fold increase in CD8 T-cells and a 4-fold increase in CD4 T-cell count per milligram of muscle (Fig. S4C, L–O).

These findings are further supported by the characterization of the draining inguinal lymph nodes on day 4. We observed an increase in overall immune cell count in the aQIV group compared to the PBS group (Fig. 3E). The increase of immune cells consisted of macrophages, and particularly muMφs (Fig. 3F and Fig. S5A). There was also an increase in lymphocytes including Tregs (Fig. 3G and S4I), and CD19^+^ B-cells (Fig. 3H) compared to PBS group. By day 4, the counts of CD8 T-cells, CD4 T-helper cells, and neutrophils were no longer significantly elevated in the aQIV group compared to the PBS group, as seen on day 2. Although we found that the cDC2 count per milligram of muscle in the aQIV group was 3-fold higher on day 4 than on day 2, the dendritic cell and cDC2 counts per lymph node were not significantly higher in the aQIV group compared to the PBS group (Fig. S4K and S4M).

Finally, we noted that while AddaVax induces similar kinetics in the muscle at both time points but doesn’t lead to an increase in immune cells in the draining lymph nodes like the aQIV group. The immune response induced by unadjuvanted QIV was minimal across all immune cell populations in both the muscle and lymph nodes. This is consistent with our previous findings, which showed that QIV is less immunogenic, yielding a lower level of vaccine-specific antibody response compared to aQIV (Fig. 1E). These findings suggest that the muscle response following aQIV or AddaVax injections mirror the muscle regenerative pathway, beginning with a pro-inflammatory response most evident at day 2 and transitioning towards a pro-repair response by day 4 post-damage.

### aQIV injection shows a reduced macrophage and Treg recruitment in aged mouse muscles and draining inguinal lymph nodes when compared to young mice

Our hypothesis for MF59’s mechanism of action requires intact macrophage and Treg responses. It is known that age-associated immunosenescence dampens geriatric innate and adaptive immunity, in parallel with muscle regenerative capacity declining with age (Agarwal et al., 2010). Specifically, Tregs and macrophages fail to accumulate in the injured aging muscle, and show an impaired phenotype that is inefficient at resolving the inflammatory milieu (Kuswanto et al., 2016).

**Fig 4.**
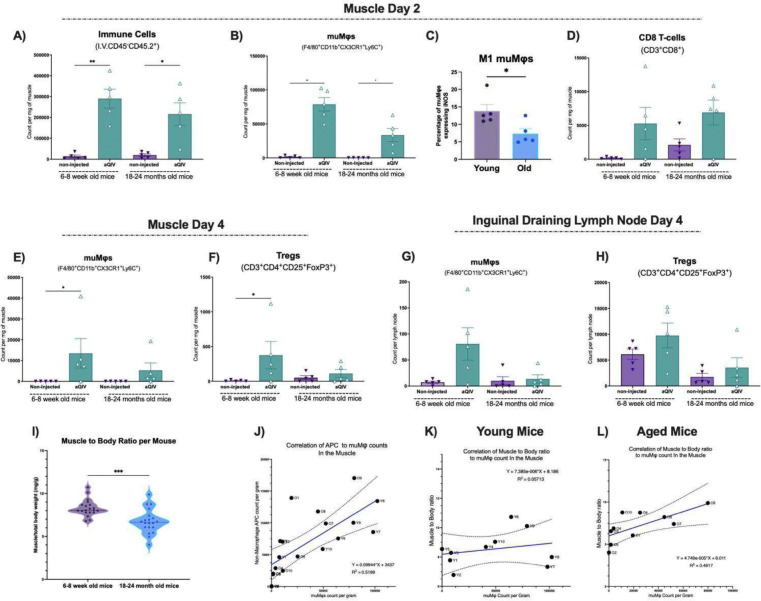
Characterization of muscle and draining inguinal lymph node immune kinetics prior to injection or after an aQIV injection in young and aged mice. Count of non-circulating intramuscular **(A)** immune cells and **(B)** CX3CR1^+^ muMφs per milligram of muscle 2 days after aQIV injection compared to baseline in young (6–8 weeks) and aged (18–24 months) mice compared to baseline. **(C)** Percentage of CX3CR1^+^ muMφs that express iNOS out of total CX3CR1^+^ muMφs. **(D)** Count of non-circulating CD8 T-cells per milligram of muscle 2 days after aQIV injection compared to baseline in young and aged mice compared to baseline. Count of non-circulating intramuscular **(E)** CX3CR1^+^ muMφs and **(F)** Tregs per milligram of muscle 4 days after aQIV injection compared to baseline in young and aged mice compared to baseline. Count of non-circulating **(G)** CX3CR1^+^ muMφs and **(H)** Tregs per draining inguinal lymph node 4 days after aQIV injection compared to baseline in young and aged mice compared to baseline. **(I)** Muscle weight in milligram per total body weight in grams of young compared to aged mice. **(J)** Correlation of intramuscular MHC II^+^ antigen presenting cell count per milligram of muscle with intramuscular CX3CR1^+^ muMφs count per milligram of muscle at 2- and 4-days post-injection with aQIV. Young mice data points labeled with a *Y* and aged mice labeled with *O*. Correlation of muscle to body ratio to CX3CR1^+^ muMφs count per milligram of muscle in **(K)** young and **(L)** aged mice 2- and 4-days post-injection with aQIV. * P ≤ 0.05 ** P ≤ 0.01 *** P ≤ 0.001 **** P ≤ 0.0001 (Mann-Whitney test).

In order to investigate whether the age-associated impairment of macrophage and Treg recruitment occurs in the aQIV injected muscles and whether this affects the quality and magnitude of the response to aQIV, we first intramuscularly injected aged (18–24 months old) mice with aQIV. We then collected injected muscles with their proximal inguinal lymph nodes on days 2 and 4 post-injection and characterized their immune content using spectral flow cytometry. We compared the aged muscles and draining inguinal lymph nodes after injection and at baseline with young (6–8-week-old) mice. The total immune cell count per milligram was comparable between the young and aged injected muscles on day 2 (Fig. 4A). The increase in numbers of eosinophils and neutrophils per milligram of muscle were largely preserved across both age groups on day 2 (Fig. S6A and S6B). Meanwhile, we found a 2-fold decrease in muMφs count per milligram of muscle in aged mice compared to the young mice (Fig. 4B). The infiltrating muMφs macrophages polarized less towards an M1 phenotype; iNOS^+^ muMφs counts decreased by 2-fold in aged mice compared to young mice_(Fig. 4C). This suggests that recruitment of macrophages to the injured muscle and their subsequent activation towards a pro-inflammatory state are both impaired in aged hosts. The CD8 T-cell response seen on day 2 was largely preserved between young and aged mice, consistent with age-associated changes to muscle regeneration described in the literature, which state that CD8 T-cell recruitment is largely unaffected by age (Sousa et al., 2024) (Fig. 4D). These findings show that the macrophage pro-inflammatory response is differentially affected by aging when compared to other immune cells present at this early stage.

On day 4, a time point associated with the muscle immune niche pivoting towards pro-repair cells and phenotypes, we found a 3-fold decrease in muMφs and Tregs in the aQIV injected muscles of aged mice compared to the young mice (Fig. 4E and 4F). We also did not detect an increase in muMφs and observed reduced Treg count in the aged mice draining inguinal lymph nodes on day 4, in contrast with results in young mice (Fig. 1G and 1H). The impairment of muscle regenerative response is associated with muscle mass loss and a decrease in muscle quality, all of which are more prevalent in aged hosts. We found that the aged mice had a reduced muscle to body ratio compared to younger mice, indicative of age-associated muscle atrophy (Fig. 4I) (Goodpaster et al., 2006). Collectively, these data demonstrate that there are marked changes in the aged muscle immune environment in response to adjuvanted vaccination, in clear contrast with what happens in young mice.

To better understand the role of the muMφ population in vaccine response, and the effects of their age-associated reduction in the milieu, we investigated the correlation between muMφs count with the count of total muscle MHC II^+^ non-macrophage antigen presenting cells (APCs) on days 2 and 4 in young and aged mice. We found a significant positive correlation between the two variables that can be represented with a linear regression with a slope of 0.1 and an R^2^ value of 0.52. This suggests that there is a positive association between the number of muMφs and the number of APCs in both age groups (Fig. 4J).

Some of the aged mice showed a muscle weight to body weight ratio within the range of young mice, so we investigated whether the ratio could predict the magnitude of the intramuscular macrophage response. We found a statistically significant positive correlation between muscle to body ratio and macrophage response in the aged mice, but not in the young mice (Fig. 4K and 4L). This highlights that muscle health correlates with the macrophage response, and could also be linked to the degree of antigen presentation and quality of the subsequent vaccine response.

**Fig 5.**
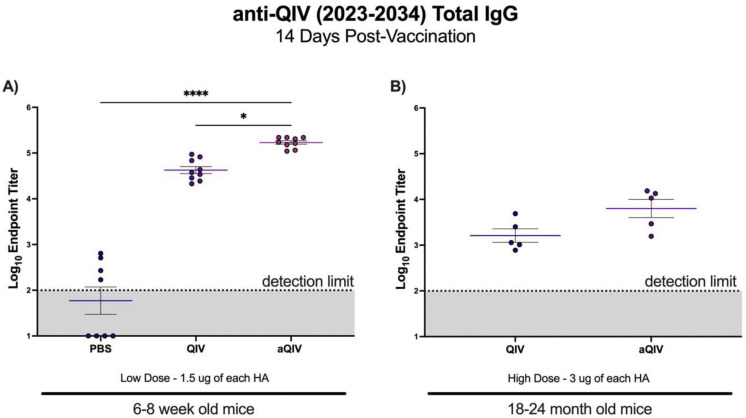
Vaccine-specific antibody titers are reduced in aged mice after QIV and aQIV vaccination compared to young mice. Vaccine Specific antibody titers in serum collected 3 weeks post vaccination in **(A)** young mice vaccinated with 3 μg HA equivalent of QIV (2023–2024 season) and **(B)** aged mice vaccinated with 3 μg HA equivalent of QIV (2023–2024 season). * P ≤ 0.05 ** P ≤ 0.01 *** P ≤ 0.001 **** P ≤ 0.0001 (Kruskal-Wallis test).

To assess the quality of the adaptive immune response to vaccination, we characterized the antibody response following QIV and aQIV vaccination in aged mice compared to young mice. In case geriatric vaccines are not adjuvanted (FluAd^®^, Seqirus), older adults (>65 years) often receive a higher dose influenza vaccine (Fluzone High Dose^®^, Sanofi Pasteur) during seasonal vaccination than the standard dose for adults. To model the differences in antigen dosage received per age group in humans, we vaccinated aged mice with a high dose QIV (2023–2024 season, 3 μg HA equivalent), while young mice were vaccinated with a half dose of the high dose QIV (2023–2024 season, 1.5 μg HA equivalent). We found QIV and aQIV induced a robust vaccine-specific antibody response by 14 days post-vaccination in young mice. aQIV also induced a significant increase in Log_10_ antibody titers compared to young mice vaccinated with QIV alone, indicating there was an adjuvant effect from the inclusion of AddaVax. In aged mice, paralleling previously described impaired immune recruitment to the muscle and lymph nodes, QIV and aQIV vaccine-specific antibody titers were reduced by 30% compared to young mice, despite being vaccinated with a higher dose of QIV. No significant difference in antibody titer was observed between QIV and aQIV formulations in aged mice (Fig. 5A and 5B).

### Adoptive transfer of young bone marrow-derived macrophages to aged mice can rescue the macrophage response to aQIV in the injected muscle and draining lymph nodes and induces higher levels of Th2-associated cytokines in the lungs upon influenza infection.

To further understand the contribution of the muscle macrophage response to the quality of the vaccine response in aged mice, we optimized a mouse model that allows us to deliver bone marrow-derived macrophages (BMDMs) into the muscle. We first validated the best route of administration to maximize the number of BMDMs localizing to the injected muscle without inducing further muscle damage. To achieve this, we injected young mice either intravenously (IV) or intramuscularly (IM) in the quadriceps with 300,000 CellTrace^™^-labeled live BMDMs cultured from young female mice of identical age, and strain (yBMDMs). One day later, we vaccinated the mice with PBS or aQIV in the quadriceps muscles. 4 days later we harvested the blood, lungs, muscles, spleens, and inguinal lymph nodes to characterize the immune cell populations using spectral flow cytometry. In the IM transfer group, we found no labeled BMDMs in any of the sampled organs other than the injected muscles. yBMDMs retained in the aQIV injected muscle, unlike the PBS group. This suggests a role for adjuvant and or antigen for IM transferred macrophage retention in the muscle niche (Fig. S7A). In the IV transfer group, we found that yBMDMs are only present in the aQIV injected muscle at around an average two-fold higher than that of the IM group (Fig. S7B). In both groups, the yBMDMs were half of the overall macrophage response (Fig. S7C). Injecting the muscles directly with yBMDMs led to 28-fold increase in neutrophil count and 21-fold increase in eosinophil count in the muscles that were then injected with PBS compared to non-injected muscles from IV delivery group. Neutrophil and eosinophil recruitment to the muscle is indicative of damage, showing that IM transfer of yBMDMs is potentially inducing muscle injury (Fig. S7D). We decided to move forward with the IV delivery method since it does not require an additional IM injection that could cause additional muscle injury and since it yields a high number of recruited yBMDMs into the aQIV injected muscle.

We next delivered yBMDMs intravenously into strain- and sex-matched aged mice one day before aQIV vaccination. We harvested the muscles and draining lymph nodes to characterize the immune cell populations using spectral flow cytometry on days 2 and 4 post-vaccination. We were able to differentiate host macrophages from yBMDMs in the muscle immune niche by labeling yBMDMs with CellTrace^™^ Blue (Fig. S8 and S9). We also labeled QIV with Alexa Fluor 488, which allowed us to track binding to vaccine antigen by cells in the muscle.

**Fig 6.**
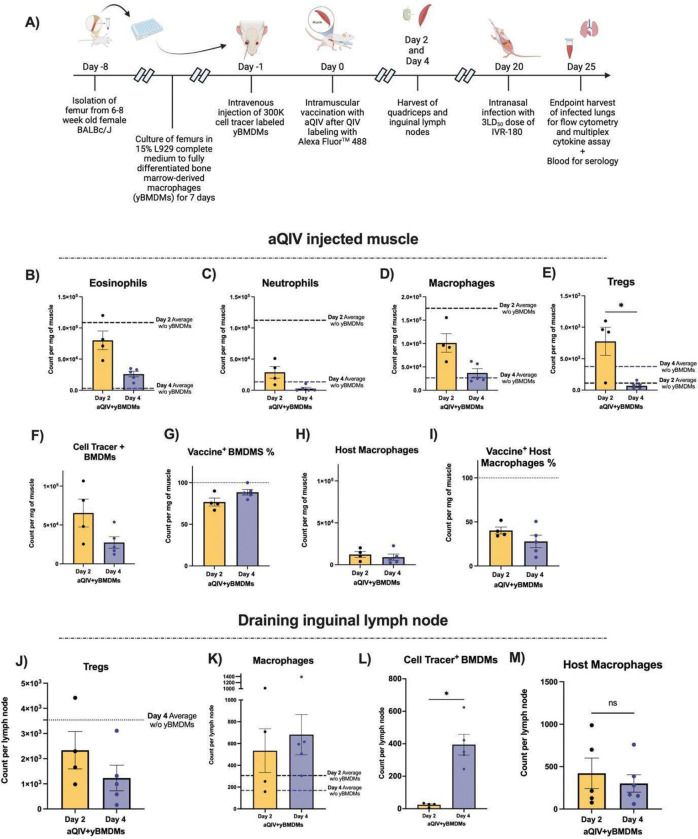
Characterization of intramuscular and draining inguinal lymph node immune kinetics post-vaccination AddaVax adjuvanted Alexa Fluor 488^™^ labeled QIV. **(A)** Experimental schematic. Count of non-circulating **(B)** Eosinophils, **(C)** Neutrophils, **(D)** macrophages, **(E)** Tregs, **(F)** Cell Tracer^+^ yBMDMs, **(G)** percentage of Vaccine^+^ yBMDMs of total yBMDMs, **(H)** host macrophages and **(I)** percentage of vaccine+ host macrophages of total host macrophages on days 2 and 4 post-aQIV injection with QIV fluorescently labeled with Alexa Fluor 488^™^ in the muscle with the dotted lines showing the previous averages of each population on days 2 and 4 post-aQIV injection and without yBMDMs delivery, as shown in Fig. 4. Count of non-circulating **(J)** Tregs, **(K)** macrophages, **(L)** CellTrace^™^ Blue^+^ yBMDMs, and **(M)** host macrophages in the inguinal draining lymph nodes on days 2 and 4 post-aQIV injection with QIV fluorescently labeled with Alexa Fluor 488^™^ in the muscle with the dotted lines showing the previous averages of each population on days 2 and 4 post-aQIV injection and without yBMDMs delivery, as shown in Fig. 4. * P ≤ 0.05 ** P ≤ 0.01 *** P ≤ 0.001 **** P ≤ 0.0001 (Mann-Whitney test)

We found that the yBMDMs, similar to muscle macrophages in young mice, are only recruited into the aQIV injected muscles and are not detected in the lungs, blood, spleen or bone marrow of injected mice on days 2, 4 and 25 post-vaccinations (Fig. S10). This suggests that the yBMDMs are not altering the macrophage niches in these organs and localize to the site of damage, in this case IM injection of vaccine. We found that eosinophil recruitment into the injected aQIV muscle is largely unaffected by yBMDM injection (Fig. 6B). Neutrophil recruitment on the other hand was reduced by yBMDM injection (Fig. 6C). Compared to aged mice that did not receive yBMDMs, the total count of macrophages on day 2 is reduced by around 2-fold in yBMDM-recipient mice (Fig. 6D). The Treg response on day 2 in yBMDM-recipient aged mice is increased by 7-fold compared to aged mice without yBMDM (Fig.6E). Over 75% of recruited muscle macrophages on days 2 and 4 were CellTrace^+^ yBMDMs, while the remainder was the endogenous host macrophage response. We also found that >75% of the yBMDMs were Vaccine^+^ unlike the host macrophages at days 2 and 4 post-vaccination, which were below 50% Vaccine^+^ at both time points (Fig. 6F–I). These findings suggest that yBMDMs are recruited to the muscle niche more robustly than aged host macrophages, and have a higher ability to bind to vaccine antigens.

In the inguinal lymph nodes, we saw an increase in macrophage numbers on days 2 and 4, with a 4-fold increase seen on day 4 compared to day 2. Regarding specific changes in the macrophage populations between day 2 and day 4, we saw a robust increase in yBMDMs in the draining inguinal lymph nodes on day 4 compared to host macrophages (Fig. 6J–M). We show that a singular yBMDM injection in aging mice results in a strong recruitment of macrophages to the muscle, followed by draining to the lymph nodes; adoptive transfer of young macrophages to aged hosts can rescue the macrophage recruitment dynamics seen in young mice.

**Fig 7.**
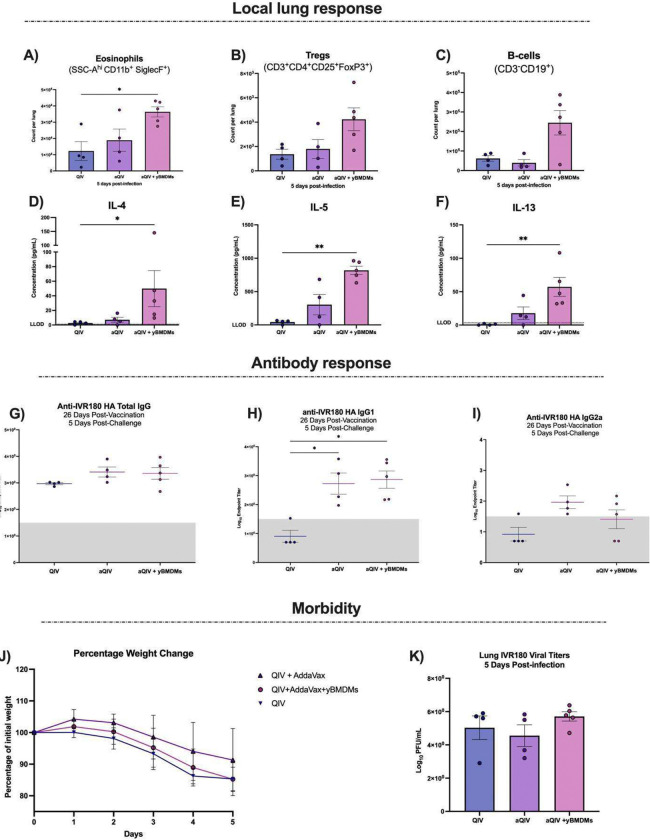
Characterization of the immune response at 5 days post-challenge with 3LD_50_ IVR-180 in aged mice vaccinated with QIV, aQIV, or aQIV+yBMDMs. Count of non-circulating **(A)** eosinophils, **(B)** Tregs, and **(C)** B-cells in the lungs 5 days post-challenge with 3LD_50_ IVR-180 aged in mice vaccinated with QIV, aQIV or aQIV+yBMDMs. Concentrations of **(D)** IL-4, **(E)** IL-5, and **(F)** IL-13 in pg/mL in lung homogenates 5 days post-challenge with 3LD_50_ IVR-180 in mice vaccinated with QIV, aQIV or aQIV+yBMDMs. **(G)** Total IgG, **(H)** IgG1 and **(I)** IgG2a IVR-180 HA specific Log10 endpoint titers 5 days post-challenge or 26 days post-vaccination with QIV, aQIV and aQIV+ yBMDMs **(J)** Percent weight change in mice vaccinated with QIV, aQIV and aQIV+yBMDMs post-challenge with 3LD_50_ IVR-180. **(K)** IVR-180 lung viral titers 5 days post-challenge with 3LD_50_ IVR-180 in mice vaccinated with QIV, aQIV and aQIV+yBMDMs. * P ≤ 0.05 ** P ≤ 0.01 *** P ≤ 0.001 **** P ≤ 0.0001 (Mann-Whitney test)

To understand the effects of the rejuvenated macrophage response on the quality of the vaccine response and vaccine efficacy, we challenged aged mice that received QIV, aQIV, or aQIV+yBMDMs 20 days post-vaccination with a 3 LD_50_ lethal dose (500 PFU per mouse) of IVR-180 H1N1 influenza A virus (Fig. 7A). We collected the lungs 5 days post-infection and characterized the immune cell composition using spectral flow cytometry, as well as the cytokine and chemokine environment using multiplex cytokine assay. Mice that received aQIV+yBMDMs had a 3-fold increase in eosinophils and Tregs, a 4-fold increase of B-cells compared to the QIV group (Fig. 7A–C). This was accompanied by a significant increase in IL-4, IL-5 and IL-13 concentrations in the aQIV+yBMDM group compared to QIV alone which is absent in the aQIV group (Fig. 7D–F).

Since mucosal influenza specific humoral response provides advanced protection by neutralizing viral particles (Ekiert & Wilson, 2012), we investigated whether the adoptive transfer of yBMDMs affects humoral response. To do so we characterized the IVR-180 HA-specific total IgG, IgG1, and IgG2a antibody binding titers by ELISA. All vaccinated groups, irrespective of adjuvant or adoptive transfer, mounted IVR-180 HA-specific antibody titers. Adjuvanting with AddaVax did not lead to an increase in IVR-180 HA-specific antibody titers, contrary to what we observed in young mice (Fig. 1E and 7G). This confirms that host age impacts the magnitude of the humoral response, in line with literature (Brydak et al., 2000). The aQIV+yBMDMs group also did not show an increase in total IgG response, suggesting that lower age-associated antibody responses cannot be reversed solely by rejuvenating the macrophage response (Fig. 7G). We found an overall decrease in IgG1 response with age, where the majority of the QIV group had no detectable IVR-180 HA-specific IgG1 titers (Fig. 7H). Adjuvanting with AddaVax resulted in all mice in the aQIV group to have detectable IVR-180 HA-specific IgG1 titers. However, similar to observations for total IgG, we also did not find a robust increase in the IVR-180 HA-specific IgG1 response in the aQIV+yBMDMs group. We did not detect IVR-180 HA-specific IgG2a titers in most of the QIV group. We saw lower titers of IgG2a in the aQIV+yBMDMs group (3 of 5 detectable) compared to the aQIV group (5 of 5 detectable) (Fig. 7I and S12).

An enhanced Th2 response 5 days post-challenge was associated with reduced morbidity in young mice (Fig. 1B). In aged mice, however, morbidity was not reduced despite an enhanced Th2 response in the aQIV+yBMDM mice (Fig. 7J), and no differences were observed in detectable viral titers in the lungs 5 days post-infection (Fig. 7K).

These results show that the macrophage response in the muscle and draining inguinal lymph nodes after aQIV injection, previously linked to a Th2-like response, can be restored in aged mice by providing them with yBMDMs. Even though this restores Th2 cytokine responses in the lungs like that of young mice, it does not remedy other age-associated immune deficits to vaccination in aged hosts, such as the inability to mount robust humoral responses post-vaccination.

## Discussion

In this paper, we show that AddaVax (MF59 research analog) skews the QIV vaccine response toward a Th2 response in mice, characterized by an elevated IgG1 ratio and increased levels of IL-4, IL-5, and IL-13 in the lungs five days post-challenge with 3 LD_50_ IVR-180 influenza A virus at 21 days post-adjuvanted vaccination. AddaVax-adjuvanted QIV (aQIV) provided better protection against lethal influenza challenge, where mice did not show substantial weight loss and had no detectable viral titers at 5 days post-infection. aQIV-vaccinated mice also had no increase in influenza-associated inflammatory cytokines like IL-1β, TNFα, IL-2, and IL-6. These influenza-associated inflammatory cytokines increased in the PBS and unadjuvanted QIV groups (Damjanovic et al., 2011; McKinstry et al., 2019; Schmitz et al., 2005; M.-L. Yang et al., 2017). Cytokines that correlate with T-cell Th1 differentiation in the lungs, including IFNγ, IL-18, and IL-12p70 increased in the PBS and QIV groups, highlighting a potential *de novo* Th1 T-cell response to the challenge virus. This is further supported by their low ability of robust viral clearance compared to aQIV and NC99 groups that had no detectable Influenza virus in the lungs by day 5 post-infection (Alvarez et al., 2023; Brombacher, 2003). It is possible that the absence of detectable concentrations of IFNγ in the lungs of aQIV-vaccinated mice is due to the complete control of viral replication by 5 days post-challenge.

Since the MF59-adjuvanted QIV vaccine is administered exclusively to the aging population, we repeated our work and characterized the AddaVax-adjuvanted QIV (aQIV) response and protection in aged (18–24 month-old) mice. We found that the benefits of AddaVax were hindered in this age group: unlike in young mice, the total IVR-180 HA-specific antibody response in the AddaVax-adjuvanted group was not significantly increased at 26 days post-vaccination compared to unadjuvanted QIV. We also found QIV-specific antibodies to be reduced in the QIV and aQIV vaccinated aged mice compared to young mice. There was also comparable weight loss in the unadjuvanted QIV and AddaVax-adjuvanted groups. Collectively, these observations suggested that the adjuvant effect of AddaVax we observed in young mice was mostly abrogated in aged mice.

This is of concern since currently, the MF59-adjuvanted QIV (FLUAD^®^) is administered to individuals 65 years and older. It is possible that the benefits of MF59 are also diminished in aged adults, similar to how the benefits of AddaVax are diminished in aged mice. Current literature on the effects of AddaVax and other MF59 similars has been conducted mainly in young mice, although these adjuvants are frequently deployed in aged populations. In particular, aQIV’s initial response has yet to be characterized in the muscle and draining lymph nodes of aged mice (Baldwin et al., 2018). We strongly believe that investigating the muscle immune response post-vaccination or injection with adjuvant is crucial, since it is the site of injection and therefore is where the vaccine immune response initiates. One factor that ties into muscle immune responses is muscle regeneration, a process that is largely impaired in individuals of this age group (Muñoz-Cánoves et al., 2020). Systemic aging is complex and variable, but previous research has highlighted some key changes that occur with aging that largely affect the regenerative pathway (Tidball et al., 2021). These changes occur due to many factors including chronic inflammation, immune senescence, bone marrow bias towards myelopoiesis, and increases in muscle adipose tissue (Bleve et al., 2022; Cui & Ferrucci, 2020; Ross et al., 2024; Tobin et al., 2021). These factors have been shown to deregulate macrophage response. For example, M2 macrophages are more abundant in the aging muscle environment at baseline, but their response to injury is largely impaired with loss of distinct M1 and M2 responses post-injury (Reidy et al., 2019; Sousa, n.d.; Sousa et al., 2023). It has been also shown that the Treg response is largely reduced in the aging muscle environment (Keilich et al., 2023; Rocamora-Reverte et al., 2021).

To investigate the age-associated decrease in the adjuvant effect of AddaVax on QIV, we characterized the immune response to aQIV in the muscle and draining lymph nodes. In 6–8 week old mice, we found that AddaVax alone induced a regenerative-like response in the muscle, characterized by an influx of eosinophils, neutrophils, and iNOS^+^ muMφs macrophages by day 2. On day 4, the response is dominated by Arginase I^+^ muMφs and Tregs, which mirror the pro-repair aspects of a homeostatic muscle regenerative response. We also observed that the increase in muMφs and Tregs is mirrored in the aQIV draining lymph nodes by day 4. We believe that this increase in macrophages in the lymph nodes indicates potential drainage from the muscle, a phenomenon that has been previously demonstrated (Calabro et al., 2011). The count of muMφs correlated with dendritic cell counts in the muscle on day 4, and dendritic cells have been shown to play a crucial role in vaccine antigens presentation and the magnitude of their recruitment into the injection site has been shown to correlate with the magnitude of the humoral response downstream of vaccination (Takano et al., 2022). These findings suggest the importance of muscle regeneration and pro-repair macrophages in the effect of the AddaVax adjuvant. Therefore, we hypothesized that the lack of efficacy of the adjuvanted vaccine in the aging population is partly due to impairments in the muscle regenerative response, characterized by the loss of pro-repair macrophage activity and age-associated muscle atrophy (Ciabattini et al., 2018). We found that indeed, muscle and lymph node muMφs and Treg numbers are reduced in aged mice compared to young mice after adjuvanted QIV vaccination.

Pro-repair M2 macrophages express Th2 polarizing cytokines like IL-4, IL-10 and IL-13 and could be contributing to Th2 skewing of MF59-adjuvanted QIV response (Z. Yang & Ming, 2014). To investigate the importance of age on muscle macrophage response, we intravenously injected aged mice with CellTrace^™^ Blue labeled bone-marrow derived macrophages (BMDM) differentiated and cultured from isolated young mouse femurs (yBMDMs) one day before vaccination with Alexa Fluor 488 fluorescently-labeled QIV adjuvanted with AddaVax. The yBMDMs were recruited to the muscle and took up the Alexa Fluor 488^™^ labeled QIV. By day 4, the yBMDMs were observed in the draining lymph node, restoring the macrophage count in the draining lymph node to comparable levels seen in young mice. Twenty-five days post-vaccination and 5 days post-infection with a 3 LD_50_ dose of the IVR-180 influenza virus, we found that aged mice receiving yBMDMs the day before vaccination exhibited higher levels of Th2-associated cytokines in the infected lungs including IL-4, IL-5 and IL-13 similar to the profile observed in younger mice. We also found an increase in Tregs, eosinophils and B-cells infiltrating in infected lungs. The increase in eosinophils has been discussed in our previous work as potentially non-pathogenic in the context of Trivalent Inactivated Influenza Vaccine (TIV), and has been shown to be associated with an increase of Th2-associated cytokines present in the lungs post-influenza infection in vaccinated mice (Chang et al., 2023; Piehler et al., 2011). We also found a higher IgG1 to IgG2a ratio of IVR-180-specific antibodies in aged mice that received yBMDMs compared to those vaccinated with QIV or aQIV, indicative of skewing of host responses towards Th2. This shows that supplementing aged mice with yBMDMs can restore the macrophage response in aQIV-injected muscle and draining inguinal lymph nodes to the levels of young mice. Transfer of yBMDMs into aged mice also increased the Th2 response seen in the lungs to resemble that of young mice that received aQIV and then infected with IVR-180 H1N1 influenza virus. This shows that muscle macrophages, recruited for muscle regeneration, are critical players in driving Th2-skewing during AddaVax-adjuvanted QIV vaccination. This effect is strongly hindered by aging, but can be restored by supplementation of young macrophages. These findings highlight the crucial role of the regenerative macrophage response in MF59’s mechanism of action as a seasonal influenza vaccine adjuvant.

Our findings show that squalene-based adjuvants like MF59 enhance vaccine immunity through muscle regeneration, particularly through regenerative macrophages. This is important because pro-repair macrophages express Th2-associated cytokines in the late pro-repair stage of muscle regeneration like IL-4, IL-10 and IL-13 (Ruffell et al., 2009; Wang & Zhou, 2022). Therefore, the Th2 skewing seen in aQIV could be driven by pro-repair macrophages. This highlights the importance of connecting literature studying muscle regeneration and muscle immunity with intramuscular vaccine responses since it is the predominant site of injection for most major clinical vaccines. The muscle regenerative pathways can be altered by diabetes (Espino-Gonzalez et al., 2024) or the microbiome (Mann et al., 2022), and lifestyle (Machida & Booth, 2004). Also, these results indicate that the initial response to AddaVax-adjuvanted QIV occurs over multiple days, emphasizing the need for longitudinal studies to fully understand MF59’s mechanism of action rather than assessing one time point. Furthermore, the dendritic response which is crucial for appropriate vaccine response is part of the muscle regenerative response but the draining of dendritic cells to proximal lymph nodes is dependent on QIV presence (Alvarez et al., 2008). This suggests that DCs can be imprinted by the muscle regenerative milieu as they correlate with macrophage recruitment, and understanding their role in the context of regeneration and therefore vaccine response is crucial.

We also make a novel observation that Tregs, which are crucial for appropriate muscle regeneration, overlap with the B-cell response downstream of aQIV in the draining lymph nodes (Fig. 3D, and 3G). Tregs enhance peripheral tolerance and specifically inhibit Th1 responses to prevent autoimmunity (Joller et al., 2014). Tregs can also affect humoral response drastically by modulating both T-cells and B-cells in favor of preventing a pathological response to foreign antigens (Likuni et al. 2009). Tregs are yet to be investigated as players in MF59’s mechanism of action. Characterizing Tregs post-MF59 adjuvanted QIV will allow us to understand whether they also play a role in Th2 skewing and whether inhibiting them would be favorable for a more robust humoral response to vaccination.

Furthermore, we show that aQIV’s response is impaired in aged mice consistently with the impairing of muscle regeneration. This is novel as MF59’s mechanism has not been shown to depend on muscle health previously. In aged mice using the muscle weight to body weight ratio, could be used to predict the magnitude of AddaVax’s response in the muscle and draining lymph nodes. We also show that the reduced aQIV response is due to macrophage age-associated senescence and targeting macrophages alone can restore aQIV’s Th2 response.

Our work provides tools to further investigate the mechanism of action of clinical squalene-based adjuvants, and provides a toolbox to incorporate muscle regeneration as a factor in intramuscular vaccine response. Our work, although limited to mice, can be translated to humans using clinical markers of muscle injury like serum creatine kinase, and lactate dehydrogenase levels which can become predictive markers of squalene-adjuvanted vaccine efficacy in the aging population.

## Methods

### Mice

BALBc/J mice 4–10-week-old female mice were purchased from Jackson Laboratory. For the aging experiments, Jackson laboratory purchased mice were aged in house to 18–24 months old. All mice were housed in our animal facilities at Icahn School of Medicine at Mount Sinai. All experiments were performed under protocols approved by Icahn School of Medicine at Mount Sinai’s institutional Animal Care and Use Committee.

For vaccinations, injection sites were shaved, and injected with 50 μL of 1:1 diluted seasonal or Fluzone High-Dose ^®^ (2021–2022 and 2023–2024) quadrivalent inactivated influenza vaccine (QIV) in sterile PBS. For Adjuvanting QIV with AddaVax, aQIV included a 1:1 diluted AddaVax and identical amount of diluted QIV. Mice were injected using a 31G sterile syringe. For the young bone marrow-derived macrophages, mice were anesthetized using isoflurane, and 50 μL of PBS containing around 300,000 live filtered bone marrow derived macrophages were injected into their retro-orbital sinus for intravenous delivery. For viral infection, mice were anesthetized using isoflurane, and infected with 50 μL of 3xLD_50_ of IVR-180 (A/Michigan/45/2015, H1N1) intranasally.

### Flow Cytometry

The following antibodies were used for flow cytometric staining with anti-I-A/I-E (M5.114.15.2), anti-CD172a or anti-SIRPα (P84), anti-F4/80 (BM8), anti-CD11b (M1/70), anti-Ly6C (HK1.4), anti-CD19 (6D5), anti-CD8a (53–6.7), anti-CD4 (RM4–5) all from Biolegend; anti-Ly6G (1A8-Ly6g), anti-Arginase I (A1exF5), anti-CD3 (17A2), anti-CD25 (PC61.5), anti-CD45.2 (104), anti-CD11c (N418), anti-iNOS (CXNFT), anti-FoxP3 (FJK-16s) all from eBioscience^™^; anti-SiglecF (E50–2440), anti-CX3CR1 (Z8–50) all from BD; anti-CD45 (30F-11) from Cytek. All antibodies were tittered and diluted to working concentration in staining buffer. Staining buffer consisted of 10% Fetal bovine serum, and 0.1% Sodium Aside in PBS. Viability was measured using propidium iodide solution from Biolegend, or fixable viability dye 520 from eBioscience diluted according to manufacturer instructions. Cellular fixation and permeabilization was performed using BD Cytofix/Cytoperm^™^ Fixation/Permeabilization Kit as per manufacturer’s instructions. FoxP3 intranuclear staining was performed using eBioscience^™^ Foxp3 / Transcription Factor Staining Buffer Set as per manufacturer’s instructions. Sample blocking was performed prior to surface and intracellular staining for 10 minutes using Purified Rat Anti-Mouse CD16/CD32 from BD Pharmingen^™^. Surface staining and intracellular staining were performed for 20 minutes at room temperature in the dark and were following by two washes with 200 mcl of staining buffer. Vaccine was labeled using Alexa Fluor^™^ 488 protein labeling kit as per manufacturer’s instructions. Bone marrow-derived macrophages were labeled using CellTrace^™^ blue cell proliferation kit, for flow cytometry as per manufacturer instructions.

### Cell Isolations

Quadriceps, and lungs were resected and processed using the gentleMACS tissue dissociator with the 37_M_LDK_1 protocol and in a digestion buffer containing 1 mg/mL Collagenase Type II from Thermofisher, and 25 ng/mL of DNase I from StemCell technologies in Corning^™^ Dulbecco’s Modified Eagle Medium (DMEM). After digestion, the reaction was quenched using cold 0.2mM EDTA in Phosphate-Buffered Saline (PBS) and filtered through 70-micron filters to create single cell suspension. Inguinal lymph nodes were digested mechanically using a plunger and a 70-micron filter using DMEM as collection buffer. Suspensions were then pelleted at 450g for 5 minutes and plated into a V-Bottom 96-well plate for flow cytometry staining. Blood was collected through the submandibular vein into a 15 mL falcon tube containing 0.2 mL of 0.2mM EDTA in PBS, and then lysed by adding 5 mL of RBC lysis buffer containing 8.29 mg/mL of NH_4_Cl (Ammonium chloride), 1mg/mL of KHCO_3_ (Potassium bicarbonate), and 0.2 mM of EDTA.

### Bone marrow-derived macrophages

Femurs were harvested as previously described (Liu & Quan, 2015). Bone marrow was harvested from 4 to 10 week-old mice by flushing 0.5 mL of DMEM through the femurs using a 31G syringe into a 1.5 mL Eppendorf tube. They were cultured in 15% L929 conditioned media (LCM) and 10% FBS in DMEM for 7 days in ultra-low attachments plates allowing mechanical resuspension on day 7. Cells received 50% fresh media every 2 days and reached full confluency and differentiation on day 7. On day 7, cells were prepared by mechanically collecting them in 5mL cold PBS. They were washed twice by spinning them down at 450 g for 5 minutes and repeating. The cells were then resuspended for a concentration of 3×10^6^ cells per mL of PBS and were filtered through a 70-micron filter. 50 μL of cell suspension was then injected per mouse.

### Multiplex Cytokine Assay

Lungs were collected into homogenizing tubes containing 0.5 mL of PBS and 3 mm triple-pure high impact zirconium beads from Benchmark Scientific, and flash frozen through dry ice, and stored at −80°C until the assay time. The lungs were homogenized and spun down at 450g for 10 minutes. The supernatant was then collected for the multiplex cytokine assay. The multiplex cytokine assay was done Thermofisher 23-Plex, or 12-Plex kits as manufacturer’s instructions. Data were acquired on a Luminex 100/200 analyzer (Millipore) with xPONENT software (version 4.3). Data visualization and analysis were conducted using GraphPad Prism (version 9.4.1).

### Serum Collection

Blood was collected through the submandibular vein and allowed to coagulate overnight at 4 degrees Celsius. After coagulation, tubes were spun down at 450g for 5 minutes and serum was transferred to new tubes and frozen at −20 degrees Celsius until further analysis was performed.

### Vaccine or Viral specific Antibody titers

96-well flat bottom plates were coated overnight at 4°C with 100 μL of 1 μg/mL of IVR-180 HA or 1:250 diluted vaccine mixed in a coating solution of 32 mM Na2CO3 and 64 mM NaHCO3. After coating, wells were washed twice with 200 μL of PBS supplemented with Tween resuspended from PBS-TWEEN^®^ Tablets from Millipore Sigma as per manufacturer instructions. Serum was then diluted in 5% milk PBS-T and 100 μL of diluted serum was added to the plates and incubated for 1 hour. The plates were then washed twice with 200 μL of PBS-T. For total IgG detection, goat F(ab) anti-mouse IgG H&L (HRP) (ab6823) was used from Abcam. For IgG1 detection, we used goat anti-mouse IgG2a-HRP (1081–05) from SouthernBiotech as per manufacturer instructions. For IgG2a detection, we used goat anti-mouse IgG1-HRP (1071–05) from SouthernBiotech as per manufacturer instructions. Plates were then washed twice after detection, and were developed using 1-Step^™^ Turbo TMB-ELISA Substrate Solution, and ELISA Stop Solution from Thermofisher as per provider instructions.

### Lung Viral Titers Quantification

For plaque assays, 250 μL of tenfold dilutions in PBS of collected samples were incubated on confluent monolayers of MDCK cells at 37°C. After 1 hour of incubation, the inoculum was removed by aspiration and cells were overlaid with 2% oxoid agar (Oxoid, Basingstoke, UK) mixed with an equal volume of NaHCO3-buffered 2xMEM supplemented with DEAE/Dextran and TPCK-treated trypsin (1 μg/mL). Cells were incubated for 48 hours at 37°C and 5% CO2. Plaque formation was visualized by staining of cell surfaces after fixation with 4% formaldehyde (5 min at room temperature). For staining, cells were incubated with a 1/1000 dilution of post challenge mouse serum followed by incubation with 1/1000 diluted sheep anti-mouse serum conjugated to horseradish peroxidase (GE Healthcare) and addition of TrueBlue substrate (KPL—Seracare, Milford, MA, USA)

### Statistical Analysis

All statistical analysis was done on GraphPad Prism software. The data is presented as mean ± SEM and SD was provided in the text. Statistical significance was computed through one-way ANOVA or non-parametric Mann-Whitney tests. P < 0.05 was considered significant.

## Supplementary Material

Supplement 1
