# Supplementary material for: Muscle macrophage regenerative response after squalene-adjuvanted influenza vaccination drives Th2-skewed response and is reduced with age
Source: Res Sq. 2025 Jan 28:rs.3.rs-5760877. Preprint. [Version 1] doi: 10.21203/rs.3.rs-5760877/v1 (PMC11838721; doi:10.21203/rs.3.rs-5760877/v1)
Supplement: Supplement 1 [file NIHPPrs5760877v1-supplement-1.pdf]

Online Supplemental Material

Fig. S1 shows circulating T-cell activation assays using influenza antigen or PMA+I, and multiplex cytokine assay of infected lungs of mice vaccinated with QIV, aQIV, PBS or infected with NC99 sublethal dose. **Fig. S2** shows the flow cytometry gating schematic utilized to quantify the myeloid population in the quadriceps and inguinal lymph nodes**. Fig. S3** shows the flow cytometry gating schematic utilized to quantify the lymphoid population in the quadriceps and inguinal lymph nodes**. Fig. S4** shows the immune characterization of PBS, AddaVax, QIV, or aQIV injected muscles using spectral flow cytometry on days 2 and 4. **Fig. S5** shows immune characterization of inguinal draining lymph nodes of a PBS, AddaVax, QIV, or aQIV injections using spectral flow cytometry on days 2 and 4. **Fig. S6** shows immune characterization of aQIV injected muscles using spectral flow cytometry on days 2 and 4 comparing 6-8-week-old mice and 18-24-months old mice**. Fig. S7** shows immune characterization of different organs using spectral flow cytometry 4 days after injecting 6-8-week-old mice with 300K BMDMs intramuscularly (I.M. route) or intravenously (I.V. route). **Fig. S8** shows flow cytometry gating strategy used to quantify the myeloid population in the in the inguinal lymph nodes allowing to differentiate host from yBMDMs through cell tracer. **Fig. S9** shows flow cytometry gating strategy used to quantify the myeloid population in the quadricep muscles allowing to differentiate host from yBMDMs through cell tracer. **Fig. S10** shows the characterization of different organs on different days post-vaccination and quantification of yBMDMs presence and persistence per organ. **Fig. S11** shows the characterization of lung environment 5 days after $3LD_{50}$ infection with IVR-180 using multiplex cytokine assay. **Fig. S12** shows the characterization of IVR-180 HA-specific antibody response skewing 5 days post-infection with 3LD50 of IVR-180 and 26 days post-vaccination with QIV, aQIV or aQIV+yBMDMs.

Supplementary Figures

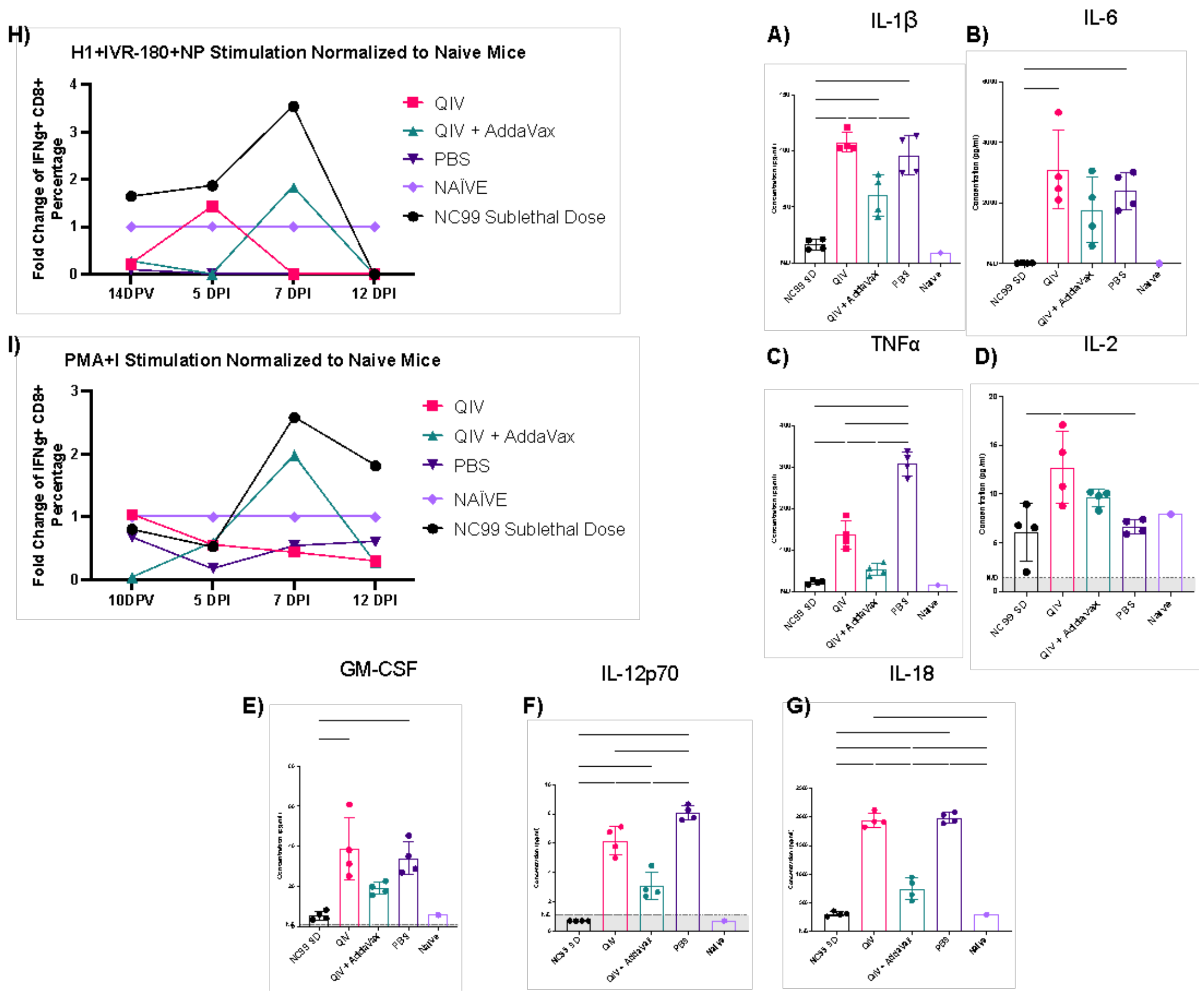

Figure S1. **AddaVax reduces lung inflammation and skews QIV response towards a Th2 response. (A-G)** Multiplex cytokine assay results showing the concentrations of IL-1β, IL-6, TNFα, IL-2, GM-CSF, IL-12p70 and IL-18 respectively in the lungs 5 days post-challenge with a 3xLD_{50} dose of IVR-180 (A/Michigan/45/2015, H1N1) **(H, I)**. Flow Cytometry data showing the fold change percentage of IFNγ+ CD8+ T-cells in the blood of the different experimental groups compared to naïve mice. * P ≤ 0.05 ** P ≤ 0.01 *** P ≤ 0.001 **** P ≤ 0.0001 (one-way ANOVA).

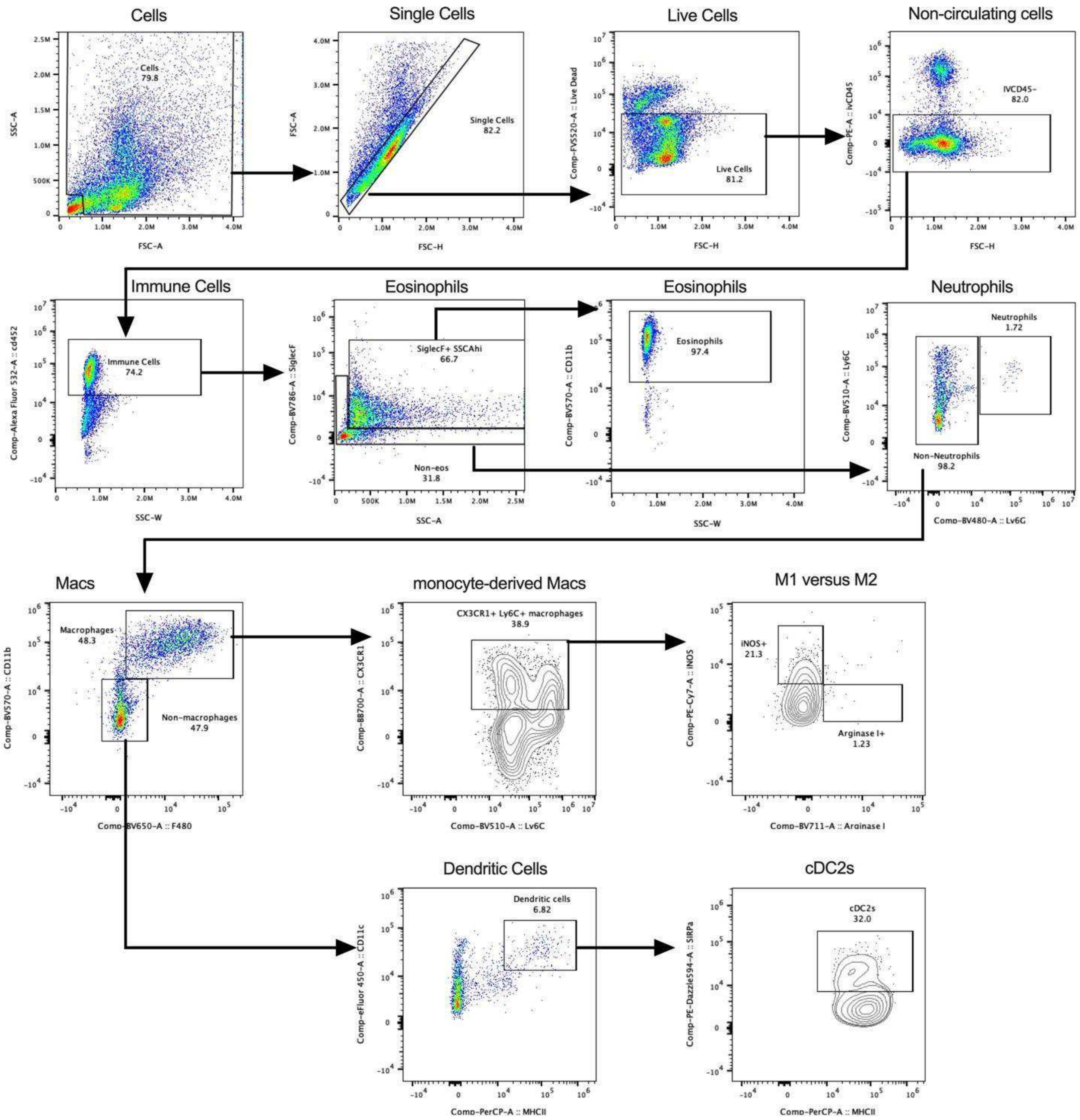

Figure S2. **Flow cytometry gating strategy used to quantify the myeloid population in the quadriceps muscles and in the inguinal lymph nodes.**

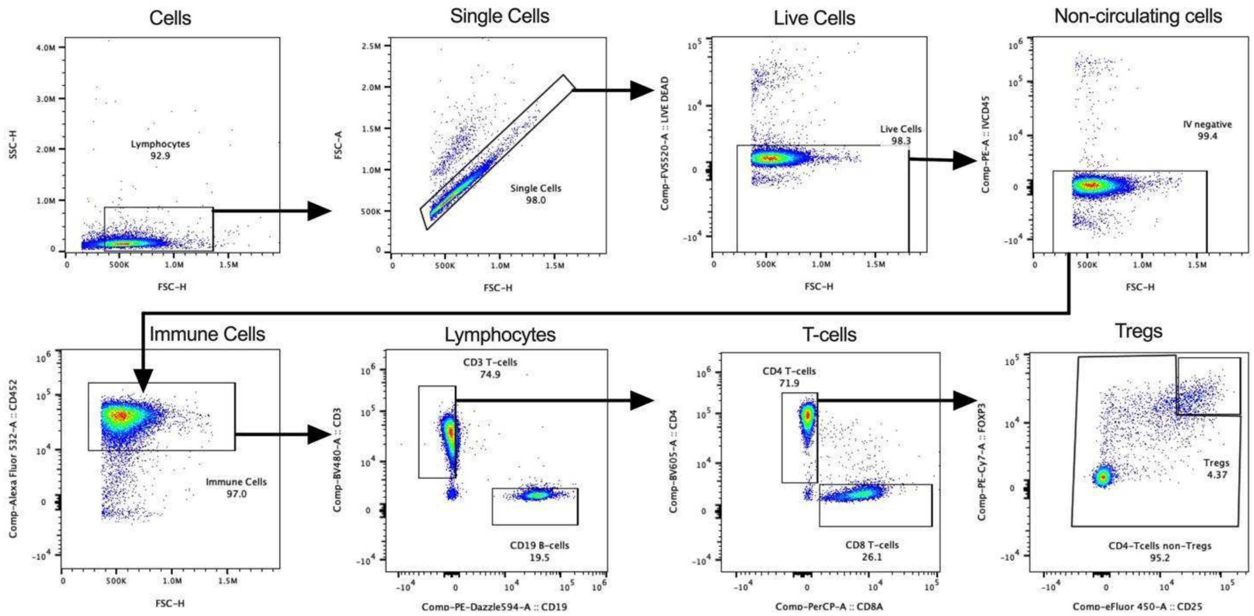

Figure S3. **Flow cytometry gating strategy used to quantify the lymphoid population in the quadriceps muscles and in the inguinal lymph nodes.**

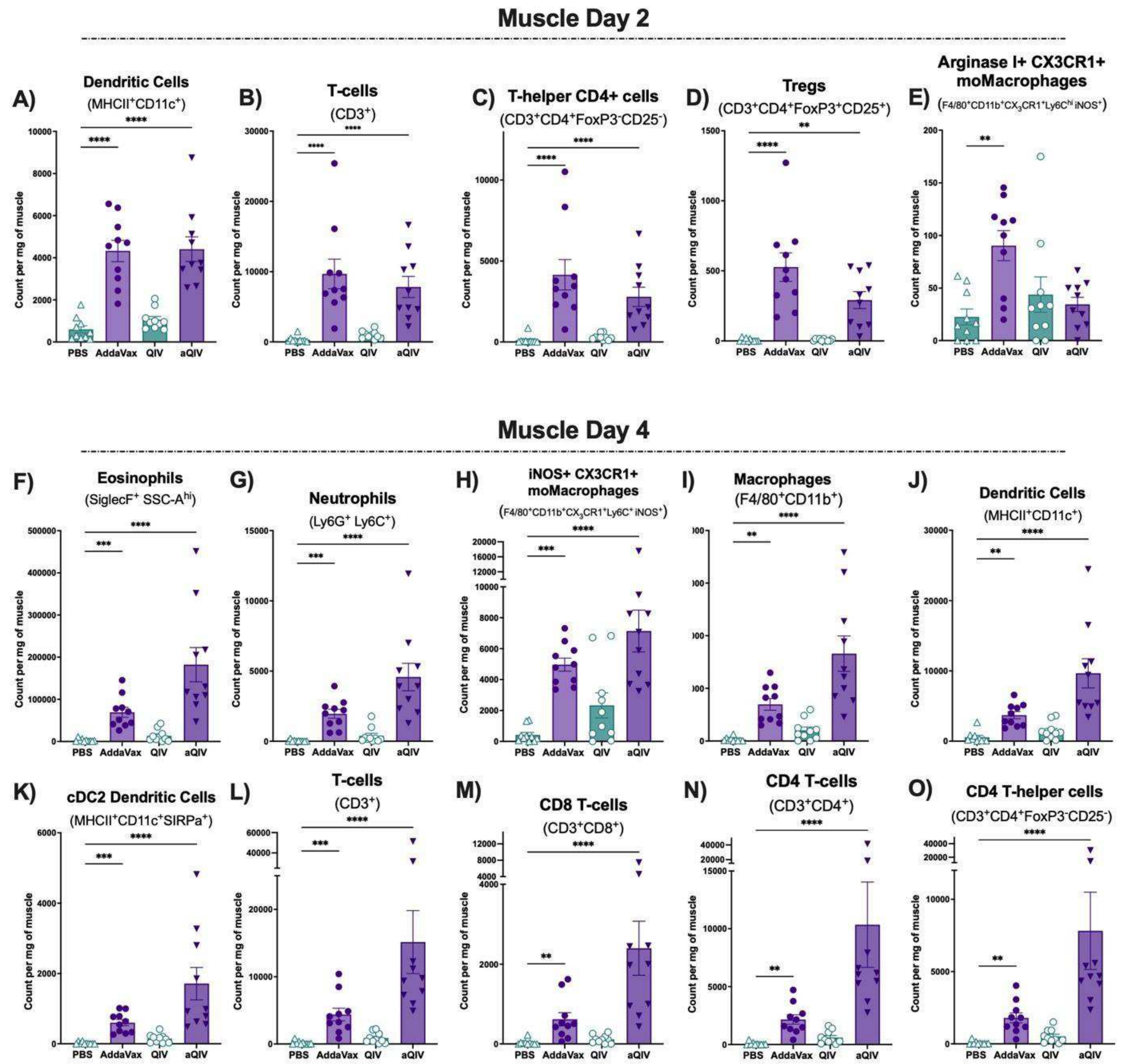

Figure S4. **Immune characterization of injected muscles using spectral flow cytometry on days 2 and 4.** Count of non-circulating **(A)** dendritic cells, **(B)** T-cells, **(C)** T-helper CD4 T-cells, **(D)** Tregs, and **(E)** Arginase I+ CX3CR1+ momacrophages per milligram of muscle 2 days post-injection with PBS, AddaVax, QIV or aQIV. Count of non-circulating **(F)** eosinophils, **(G)** neutrophils, **(H)** iNOS+CX3CR1+ momacrophages, **(I)** macrophages, **(J)** dendritic cells, **(K)** cDC2 dendritic cells, **(L)** T-cells, **(M)** CD8 T-cells, **(N)** CD4 T-cells, **(O)** CD4 T-helper cells per milligram of muscle 4 days post-injection with PBS, AddaVax, QIV, or aQIV. * $P \le 0.05$ ** $P \le 0.01$ *** $P \le 0.001$ **** $P \le 0.0001$ (Mann-Whitney test).

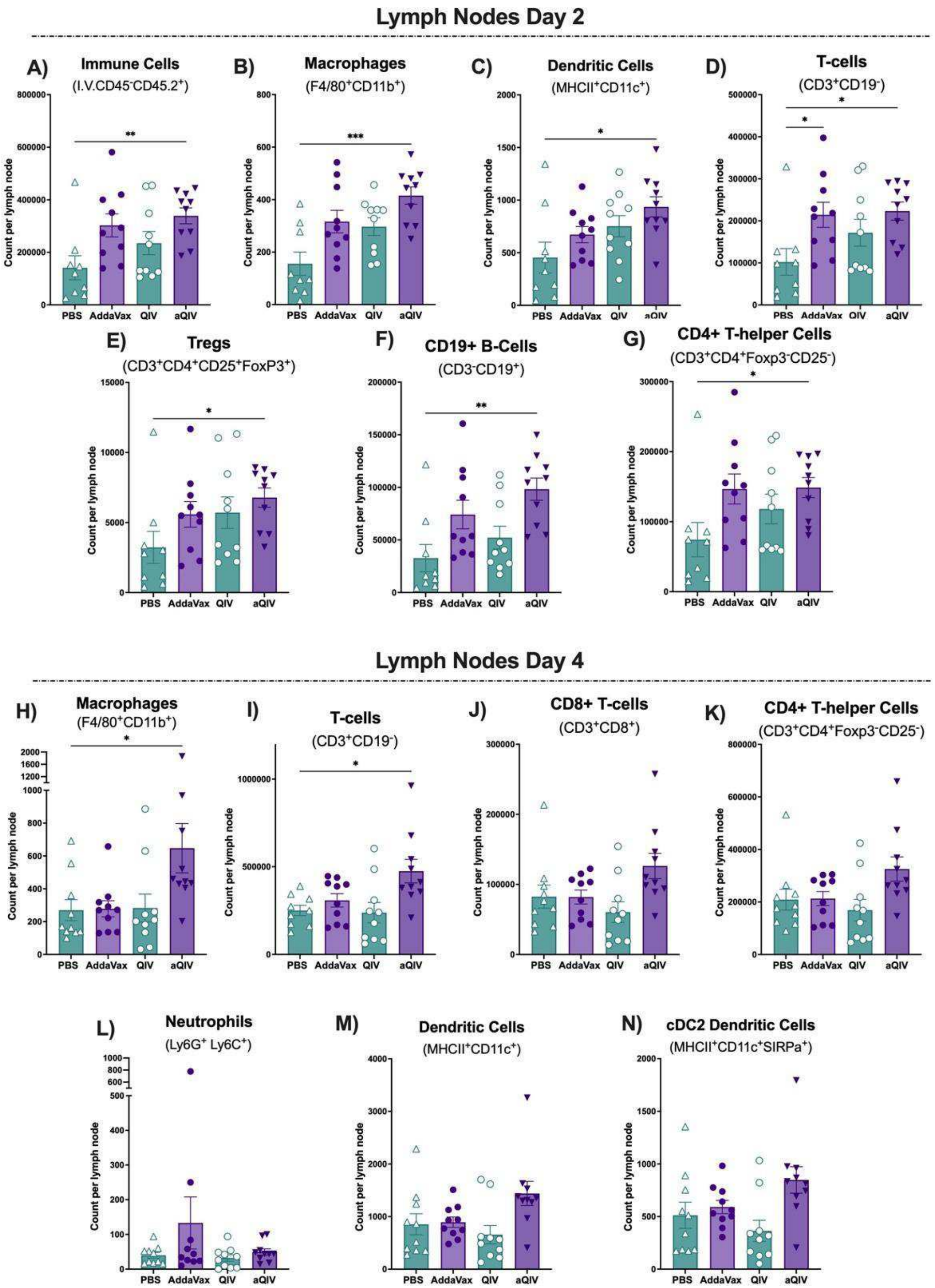

Figure S5. **Immune characterization of inguinal draining lymph nodes using spectral flow cytometry on days 2 and 4.** Count of non-circulating **(A)** immune cells, **(B)** macrophages, **(C)** dendritic cells, **(D)** T-cells, **(E)** Tregs, **(F)** CD19+ B-Cells, **(G)** CD4+ T-helper cells in the draining inguinal lymph nodes 2 days post-injection with PBS, AddaVax, QIV or aQIV. Count of non-circulating **(H)** macrophages, **(I)** T-cells, **(J)** CD8+ T-cells, **(K)** CD4+ T-helper cells, **(L)** neutrophils, **(M)** dendritic cells, and **(N)** cDC2 dendritic cells in the inguinal draining lymph nodes 4 days post-injection with PBS, AddaVax, QIV, or aQIV. * $P \leq 0.05$ ** $P \leq 0.01$ *** $P \leq 0.001$ **** $P \leq 0.0001$ (Mann-Whitney test).

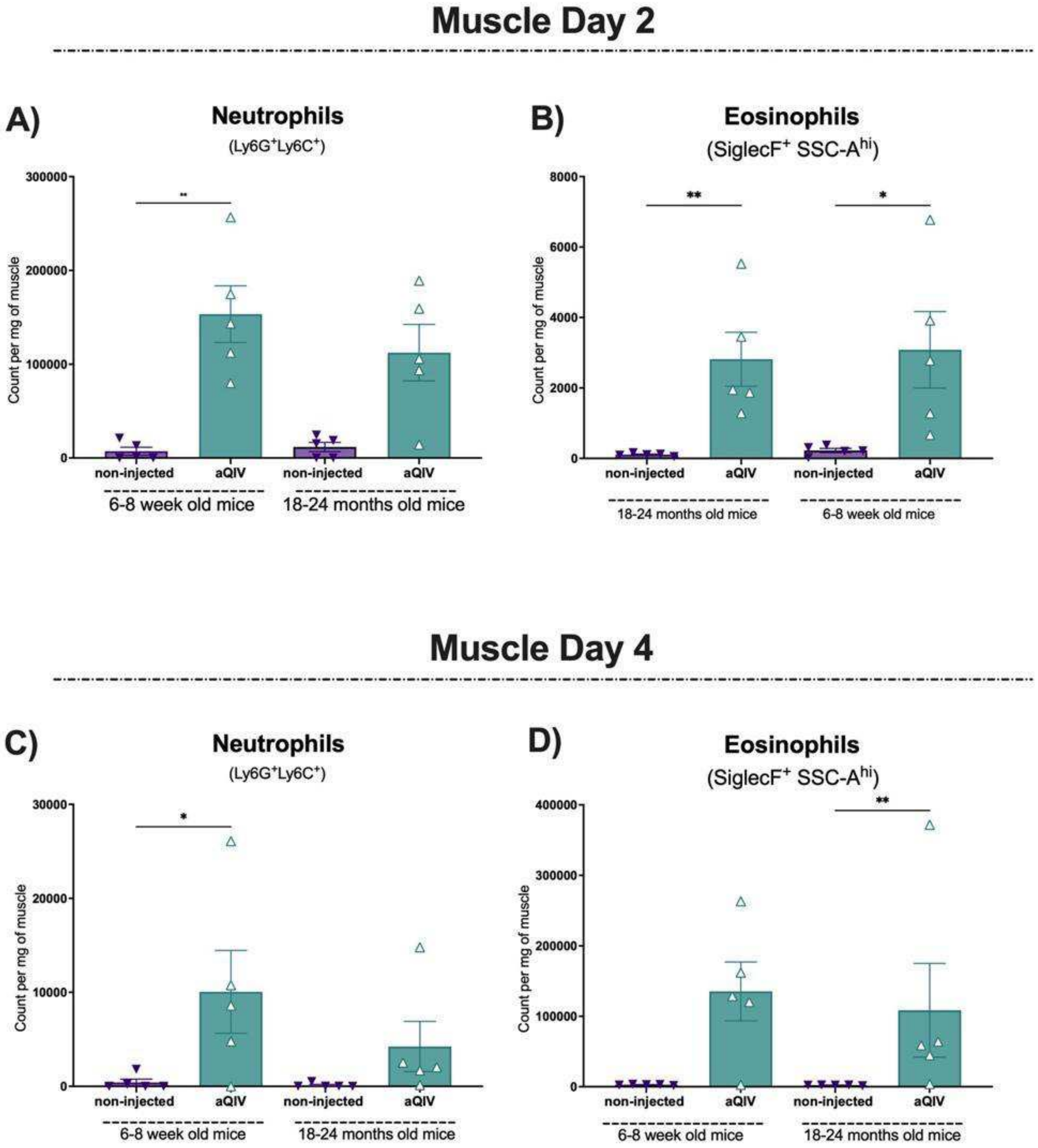

Figure S6. **Immune characterization of aQIV injected muscles using spectral flow cytometry on days 2 and 4.** Count of non-circulating **(A)** neutrophils, and **(B)** eosinophils 2 days post-aQIV injection and at baseline in 6-8-week-old and 18-24-months-old mice. Count of non-circulating **(C)** neutrophils, and **(D)** eosinophils 4 days post-aQIV injection and at baseline in 6–8-week-old and 18-24-months-old mice. * P ≤ 0.05 ** P ≤ 0.01 *** P ≤ 0.001 **** P ≤ 0.0001 (Mann-Whitney test).

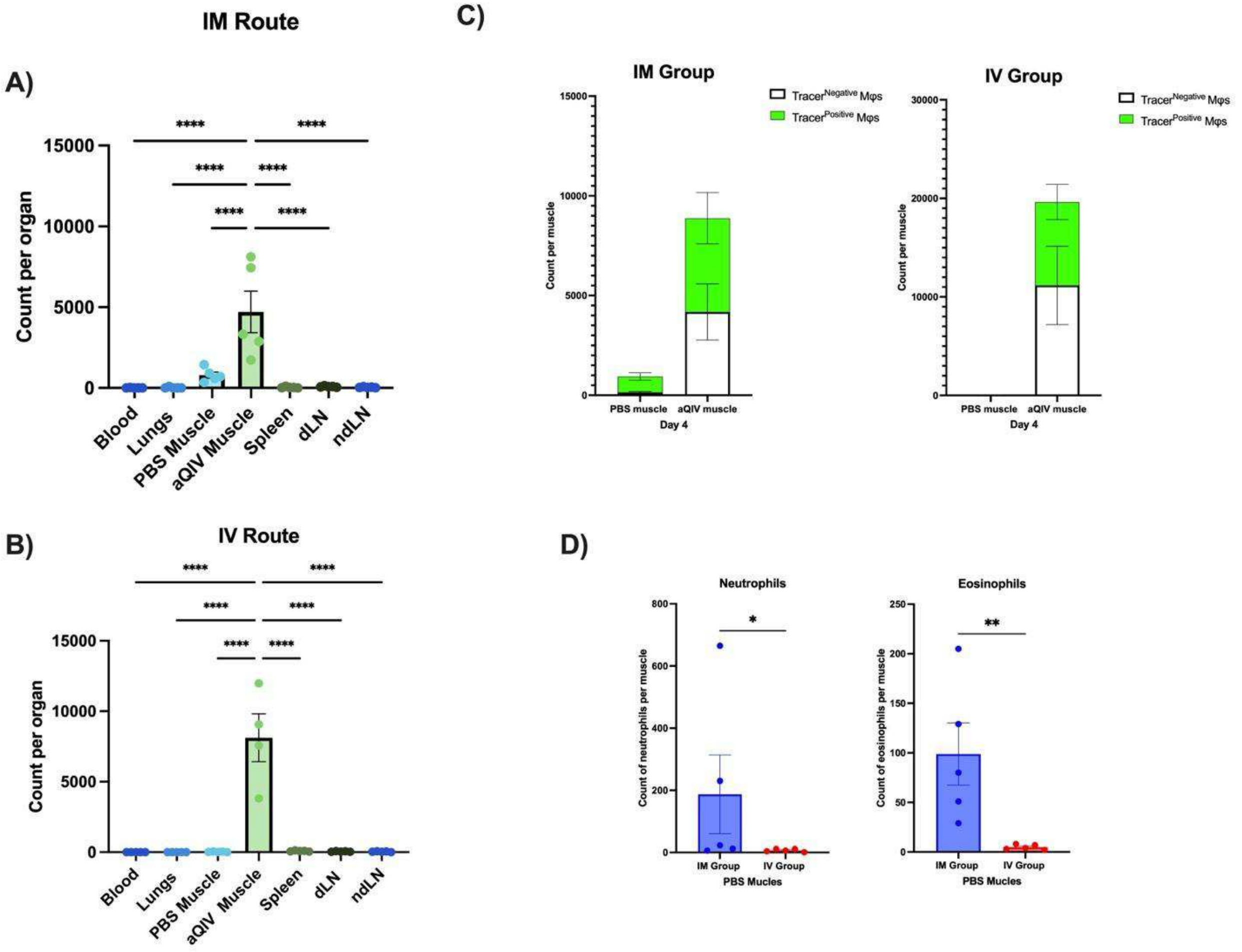

Figure S7. **Immune characterization of different organs using spectral flow cytometry 4 days after injecting 6-8-week-old mice with 300K BMDMs intramuscularly (IM group) or intravenously (IV group).** Count of macrophages that are $CD11b^{+}F4/80^{+}$ and Tracer^{+} in the blood, lungs, PBS injected muscle on day 1, aQIV injected Muscle on day 1, spleen, dLN, ndLN, of mice that received 300K live CellTrace CFSE^{+} bone marrow-derived macrophages (yBMDMs) **(A)** intramuscularly in both left and right quadricep muscles, or **(B)** intravenously on day 0**. (C)** Macrophage count and CellTrace status in PBS and aQIV injected muscles 4 days post-injection in IV and IM groups. **(D)** Neutrophil and eosinophil counts in PBS muscles of IM and IV groups 4 days post-injection. * P ≤ 0.05 ** P ≤ 0.01 *** P ≤ 0.001 **** P ≤ 0.0001 (one-way ANOVA or Mann-Whitney Test)

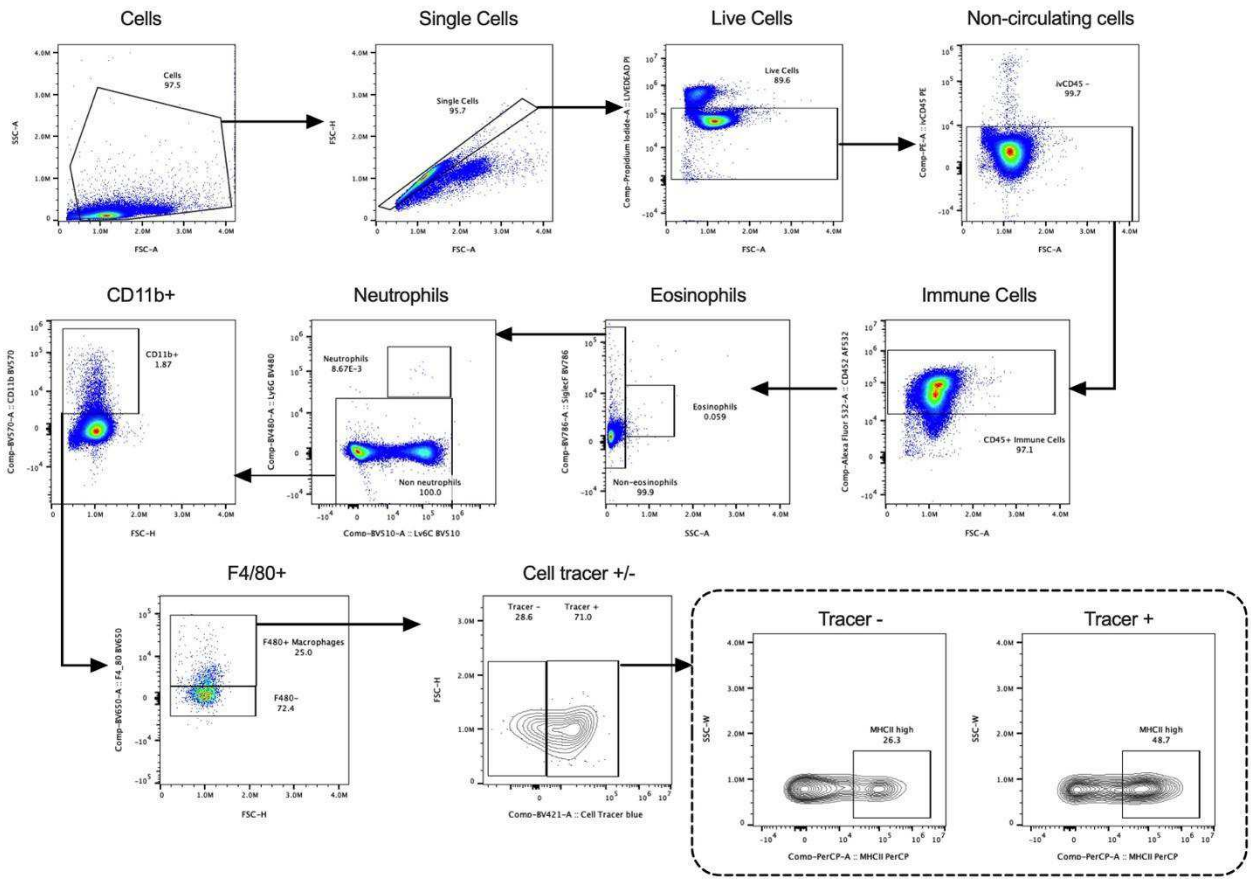

Figure S8. **Flow cytometry gating strategy used to quantify the myeloid population in the inguinal lymph nodes allowing to differentiate host from yBMDMs through cell tracer.**

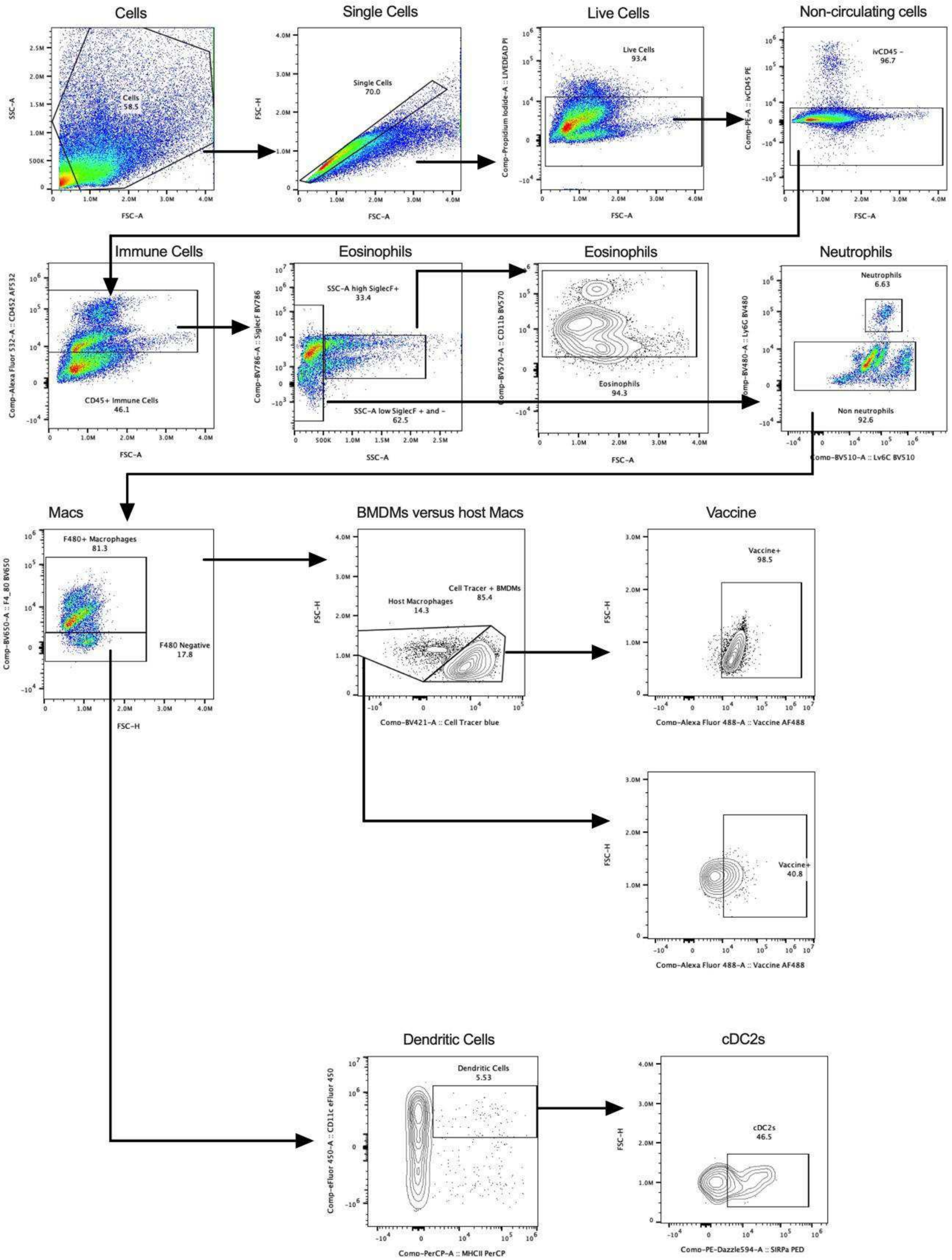

Figure S9. **Flow cytometry gating strategy used to quantify the myeloid population in the quadricep muscles allowing to differentiate host from yBMDMs through cell tracer.**

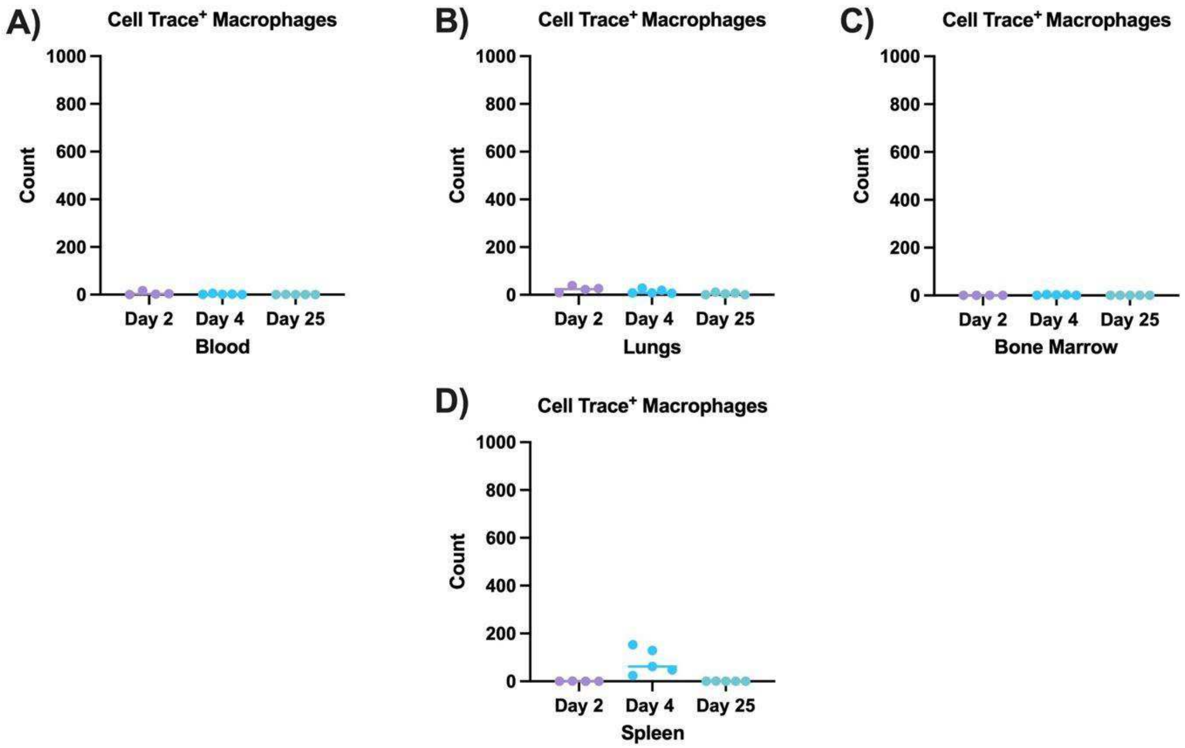

Figure S10. **Characterization of different organs on different days post-vaccination and quantification of yBMDMs presence and persistence per organ.** Count of non-circulate Cell Trace+ Macrophages in **(A)** the blood, **(B)** lungs, **(C)** bone marrow, and **(D)** spleen of mice that received a yBMDM intravenous injection on day -1, on days 2, 4 and 25 post-aQIV vaccination.

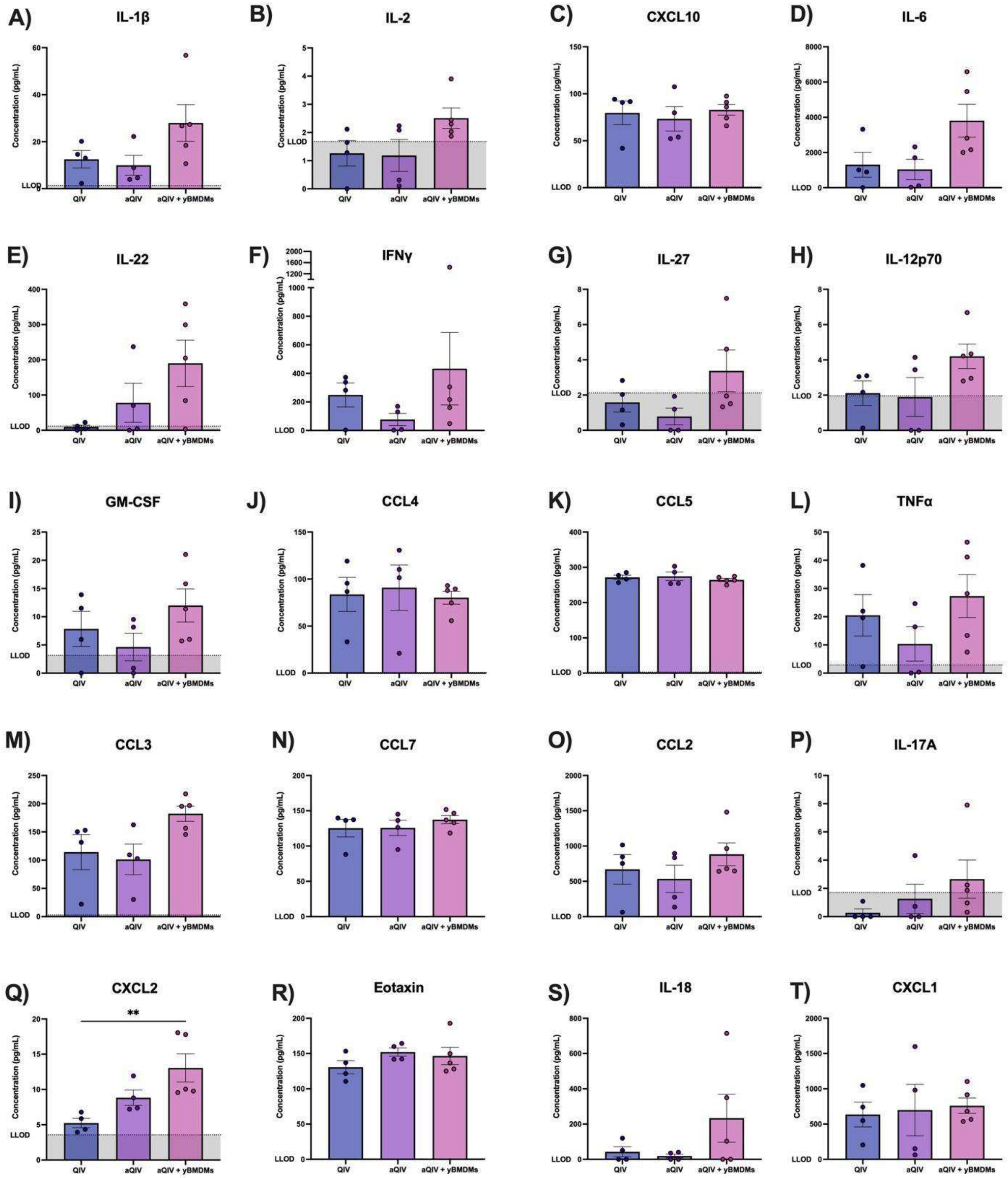

Figure S11. **Characterization of lung environment 5 days after $3LD_{50}$ infection with IVR-180 using multiplex cytokine assay.** Concentration of **(A)** IL-1β, **(B)** IL-2, **(C)** CXCL10, **(D)** IL-6, **(E)** IL-22, **(F)** IFNγ, **(G)** IL-27, **(H)** IL-12p70, **(I)** GM-CSF, **(J)** CCL4, **(K)** CCL5, **(L)** TNFα, **(M)** CCL3, **(N)** CCL7, **(O)** CCL2, **(P)** IL-17A. **(Q)** CXCL2, **(R)** Eotaxin, **(S)** IL-18, and **(T)** CXCL1 5 days post-infection with $3LD_{50}$ IVR-180 in QIV, aQIV, and aQIV+yBMDMs groups.

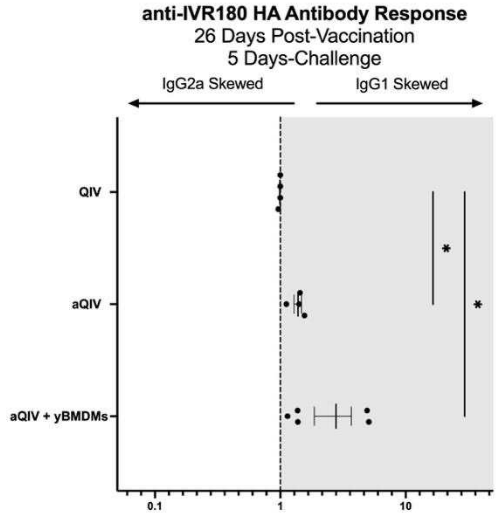

Figure S12. **Characterization of IVR-180 HA-specific antibody response skewing 5 days post-infection with 3LD50 of IVR-180 and 26 days post-vaccination with QIV, aQIV or aQIV+yBMDMs.**
